# The limited effects of social media use on misperceptions Social media’s limited role in misperceptions

**DOI:** 10.1371/journal.pone.0354486

**Published:** 2026-09-30

**Authors:** Qin Li, Robert M. Bond, Erik Nisbet, Graham Dixon, Shelly Hovick, Kilhoe Na, Duane Wegener, R. Kelly Garrett

**Affiliations:** 1 School of Communication, Ohio State University, Columbus, Ohio, United States of America; 2 School of Communication, Northwestern University, Evanston, Illinois, United States of America; 3 Merrimack College, North Andover, Massachusetts, United States of America; 4 Department of Psychology, Ohio State University, Columbus, Ohio, United States of America; National Taiwan University, TAIWAN

## Abstract

Social media use has the potential to promote misperceptions, but evidence is mixed. Despite social media characteristics that include homophilous social networks, algorithms that can reinforce biases, and social bots that promote falsehoods, there is no evidence of a strong association between social media use and misperceptions. This project offers the strongest empirical test of this relation to date, providing additional evidence of the minimal effects of social media use on misperceptions. Data come from two independent large-*N* panel studies, each paired with online tracking data, one focused on the 2020 election and the other on the COVID-19 pandemic. Within-between regression models show that social media use was not associated with a statistically significant decline in Americans’ ability to discriminate between true and false claims on any of the four platforms examined—Facebook, Twitter, YouTube, and Instagram.

## Introduction

Concerns about the harmful influence of misinformation on social media is widespread in the U.S., and some empirical findings suggest that this is warranted [1]. Social media are an increasingly common and trusted source of news [2,3], where users often cluster into politically homogeneous communities [4,5]. Within some of these networks, particularly at the fringes, false and inflammatory content circulates widely [6,7]. Platform algorithms can inadvertently promote the sharing of misinformation and can reinforce users’ biases [8–10], while coordinated activity by social bots strategically amplifies some types of misinformation [11]. Even when fact-checked, falsehoods on Twitter tend to travel farther and faster than truths, though this pattern does not replicate across all platforms [12,13].

At the same time, empirical research on social media’s role in promoting misperceptions points to a more limited set of effects, consistent with the “minimal effects” tradition in communication research [14]. Political misinformation is a tiny part of the social media ecosystem and Americans share it infrequently [6,15–17]. Early panel evidence from 2016 found no clear relation between Facebook use and political misperceptions among U.S. adults [18]. Experimental studies provide stronger causal leverage but yield similarly mixed results. A 2018 deactivation study found that users who stopped using Facebook for a month showed *declines* in the accuracy of their political knowledge [15]. A subsequent 2020 deactivation study distinguished between news knowledge (recognizing current events) and fact knowledge (distinguishing true from false claims), finding that discontinuing Facebook use was associated with reduced news knowledge but increased fact knowledge; participants who stopped using Facebook showed modestly less belief in three false claims [19]. More broadly, platform-intervention studies that manipulate feed content or ranking tend to find limited effects on political knowledge, beliefs, or attitudes [16,20]. Consistent with these patterns, recent cross-national evidence points to a similarly contingent pattern, in which associations with platform use and misinformation beliefs depend on topic, platform, trust in news, and the broader information environment [17,21].

The project described here offers some of the strongest evidence to date of the limits of the effect of social media use on misperceptions, with tests conducted in the context of both the 2020 U.S. presidential election and the COVID-19 pandemic. Combining panel surveys conducted with a large sample of Americans with digital trace data, we demonstrate that increased social media use does not have a substantively meaningful relation with misperceptions in either context. Self-reported use of YouTube and Twitter is associated with a very small, but statistically significant, *improvement* in the ability to identify true and false claims but these relations are not significant when examined using behavioral data.

## Materials and methods

This research is based on two separate panels administered by YouGov. The first panel covered the 2020 U.S. presidential campaign, with data collection spanning September 17, 2020 to November 23, 2020. These analyses use five waves of data, which were spaced approximately two weeks apart. YouGov used a sample matching approach for half the sample in an effort to ensure that this was representative sample (n = 700 at baseline); the sample for which we had behavioral data was a non-probability sample (n = 700 at baseline) and included only people who volunteered to have their web behavior tracked. The second panel covered the COVID-19 pandemic from December 9, 2020 to June 16, 2021. There were seven waves, collected monthly. This was a non-probability sample as it only included participants willing to share their web behavior (N = 1200 at baseline). These studies were approved by The Ohio State University’s Institutional Review Board (Protocols #2020B0359 and #2018B0544). Participants provided written informed consent via the survey for both studies.

We measured misperceptions using a procedure modeled closely on prior work [22]. Before each survey wave, we used NewsWhip to identify news stories that had received high levels of social media engagement in the period leading up to the survey. We then reviewed these stories to identify cases in which a central factual claim could be classified as true or false. Story veracity was established using source information, coverage by other news organizations, and published fact-checks when available. For each selected story, we drafted a short statement summarizing a central factual claim from the story, and participants rated these statements on a six-point scale anchored by “definitely true” and “definitely false.” We term these statements “belief statements” as they are used to assess participant’s beliefs about the veracity of recently circulating news. See S1 File for more details and the full set of statements.

Including both true and false belief statements allows us to distinguish between believing false claims and failing to recognize true claims. We summarize this performance using signal detection theory, with belief sensitivity capturing respondents’ ability to discriminate true from false statements [23].

This measurement approach differs from common misperception instruments that rely on fixed sets of researcher-selected false claims [24], conspiracy beliefs [25], political knowledge items [26], or experimentally selected true and false headlines [27]. Although these approaches are well suited to measuring beliefs about specific claims or general news discernment, they might be more dependent on researcher decisions about which claims are substantively important. By contrast, our approach uses recent social media engagement data to identify prominent claims circulating in the information environment immediately before each survey wave.

We collected both self-reported and behavioral measures of social media use. The former was measured in the panel survey. The latter was based on tracking data provided by YouGov. The tracking data captures both app use and websites visited on personal devices, including laptops, smartphones, and tablets. We were able to compute social media use measures for most participants in both the election panel (N = 624) and the COVID-19 panel (N = 1,010) using the tracking data. We constructed three separate, but interrelated measures of social media use. First, we computed the average total duration of time that a participant spent per day on each platform for the week (election study) or month (COVID study) during which the news stories were collected. Second, we measured the average total number of visits per day that a participant made to each platform during the story collection period. Third, we computed the average number of unique hours per day that a participant visited a social media platform during the story collection period.

We also used the panel questionnaire to measure whether participants viewed any of the social media platforms (i.e., Facebook, Twitter, and YouTube) as their most important source of news.

### Pairing panel and behavior data

We contracted with YouGov to collect two independent data sets, each composed of a multi-wave panel study paired with behavioral data. The first was conducted during the 2020 U.S. election and consisted of 5 waves of data collection conducted biweekly in the lead up to Election Day. The second focused on COVID-19 and consisted of 7 waves, collected monthly beginning in December 2020. We used social media engagement data to identify several high-profile true and false claims related to each domain, constructed a series of belief statements based on those stories, and asked respondents to assess their veracity. We used these veracity assessment batteries to create a measure of participants’ ability to distinguish true and false information using signal detection theory (SDT). In SDT, sensitivity is the ability to discriminate between signal (in our case, true statements) and noise (false statements). Sensitivity ranges from 0 (perfect mis-categorization) to 1 (perfect categorization), with 0.5 being the equivalent of chance. We use this measure to assess participants’ susceptibility to misperceptions. YouGov also provided us with consenting participants’ web-browsing and app usage history during the study, providing an unobtrusive way of capturing participants’ social media use immediately prior to each wave of the panel. We used these data to construct several media use measures for each of four social media platforms: Facebook, Twitter (as it was known at the time of data collection), YouTube, and Instagram.

## Results

### Social media use and belief sensitivity

We begin by briefly describing the patterns of social media use we observe in our panels. Fig 1 shows the empirical distributions of the number of minutes spent on Facebook, Instagram, Twitter, and YouTube for the two panel studies. As the figure shows, Facebook is the most popular platform in terms of the number of participants using the site. However, YouTube’s most frequent users spend more time on it than the most frequent users of other social media platforms.

**Fig 1:**
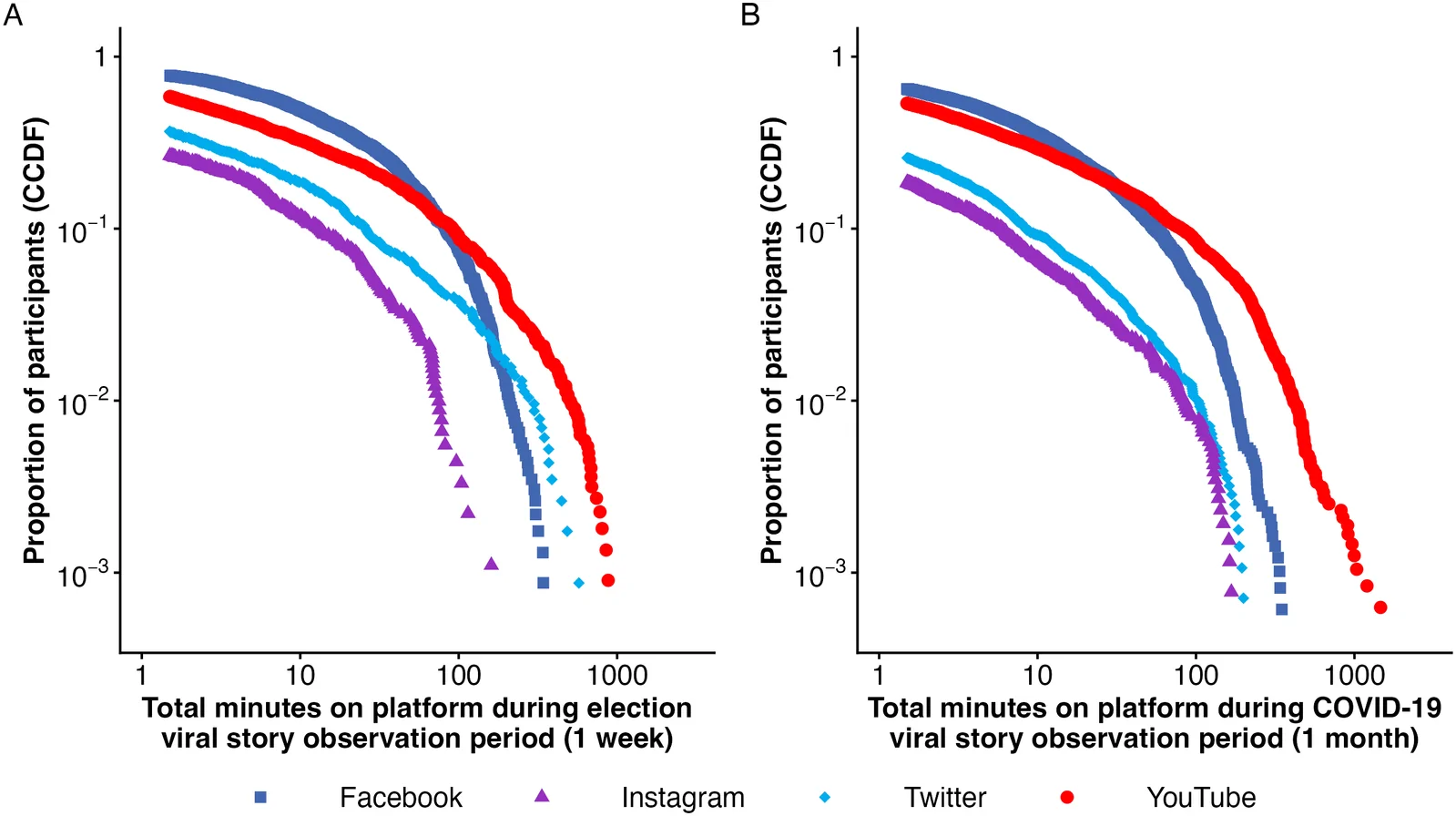
Distributions of time spent on social media platforms in two panel surveys. Distributions of minutes spent on four social media platforms across the two panel studies. Complementary cumulative distribution functions (CCDFs) of minutes spent on Facebook, Instagram, Twitter, and YouTube for panels focused on the 2020 U.S. presidential election (A) and the December 2020 through June 2021 period of the COVID-19 pandemic (B).

Consistent with prior work, simple descriptive statistics offer little evidence that Americans’ susceptibility to misperceptions is correlated with their social media use. Fig 2 uses our U.S. election panel data and shows the distributions of belief sensitivity as related to varying levels of social media use for Facebook (Fig 2A), Twitter (Fig 2B), YouTube (Fig 2C), and Instagram (Fig 2D). As shown in the figure, participants’ sensitivity averages around 0.75, and visual inspection does not show a clear relation with social media use for any of the platforms we investigate.

**Fig 2:**
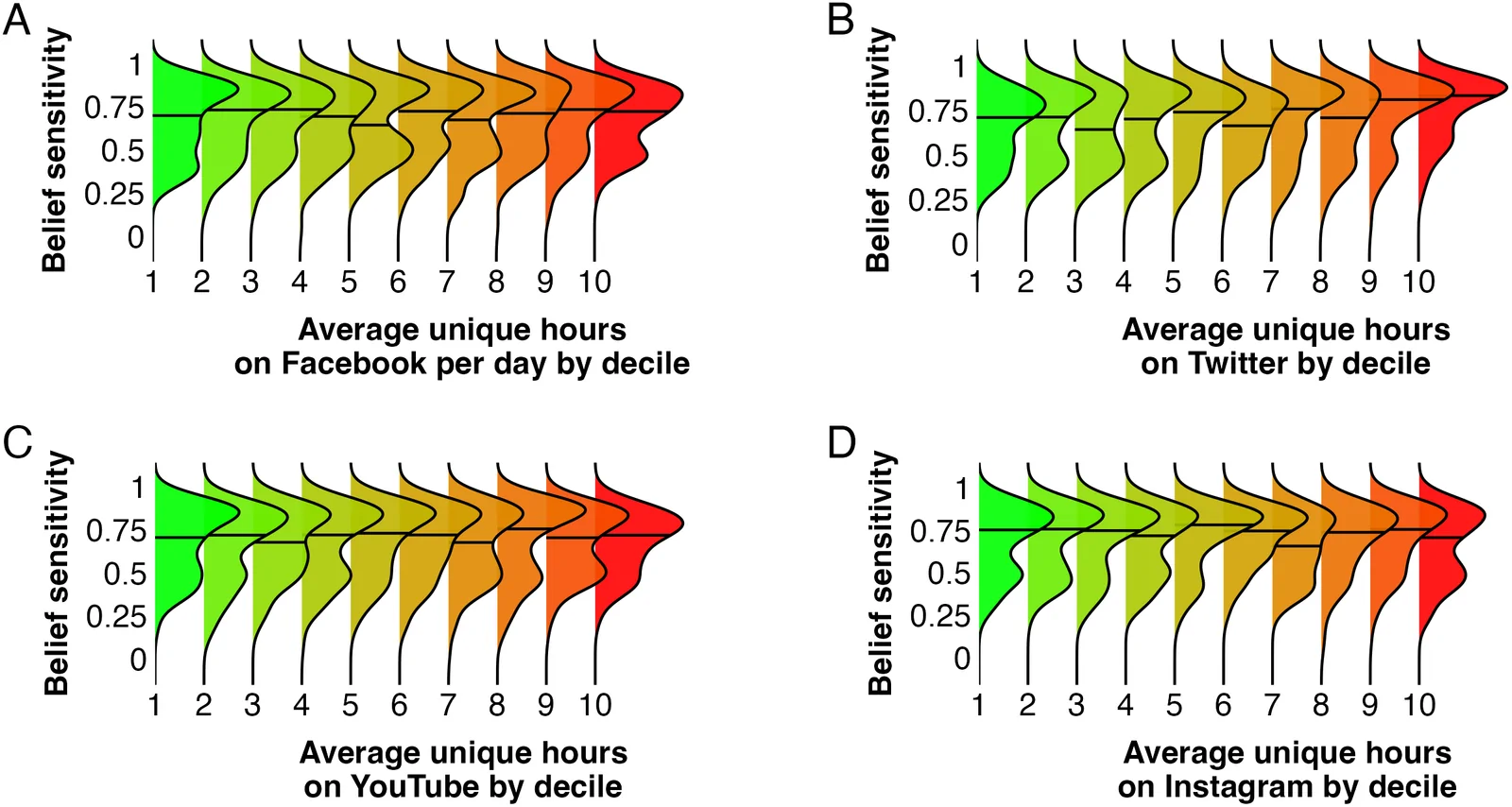
The relation between social media use and belief sensitivity related to politics. Probability density estimates of belief sensitivity in the U.S. presidential election by social media use. Panels show the decile of unique hours in the one week on (A) Facebook, (B) Twitter, (C) YouTube, and (D) Instagram during the story collection period.

Similarly, Fig 3 uses COVID-19 pandemic panel data. Again, the panel shows the distributions of belief sensitivity across the levels of social media use for Facebook (Fig 3A), Twitter (Fig 3B), YouTube (Fig 3C), and Instagram (Fig 3D). The figures are quite consistent. Again, participants’ sensitivity averages around 0.75, but the relation with social media use is unclear for all four platforms.

**Fig 3:**
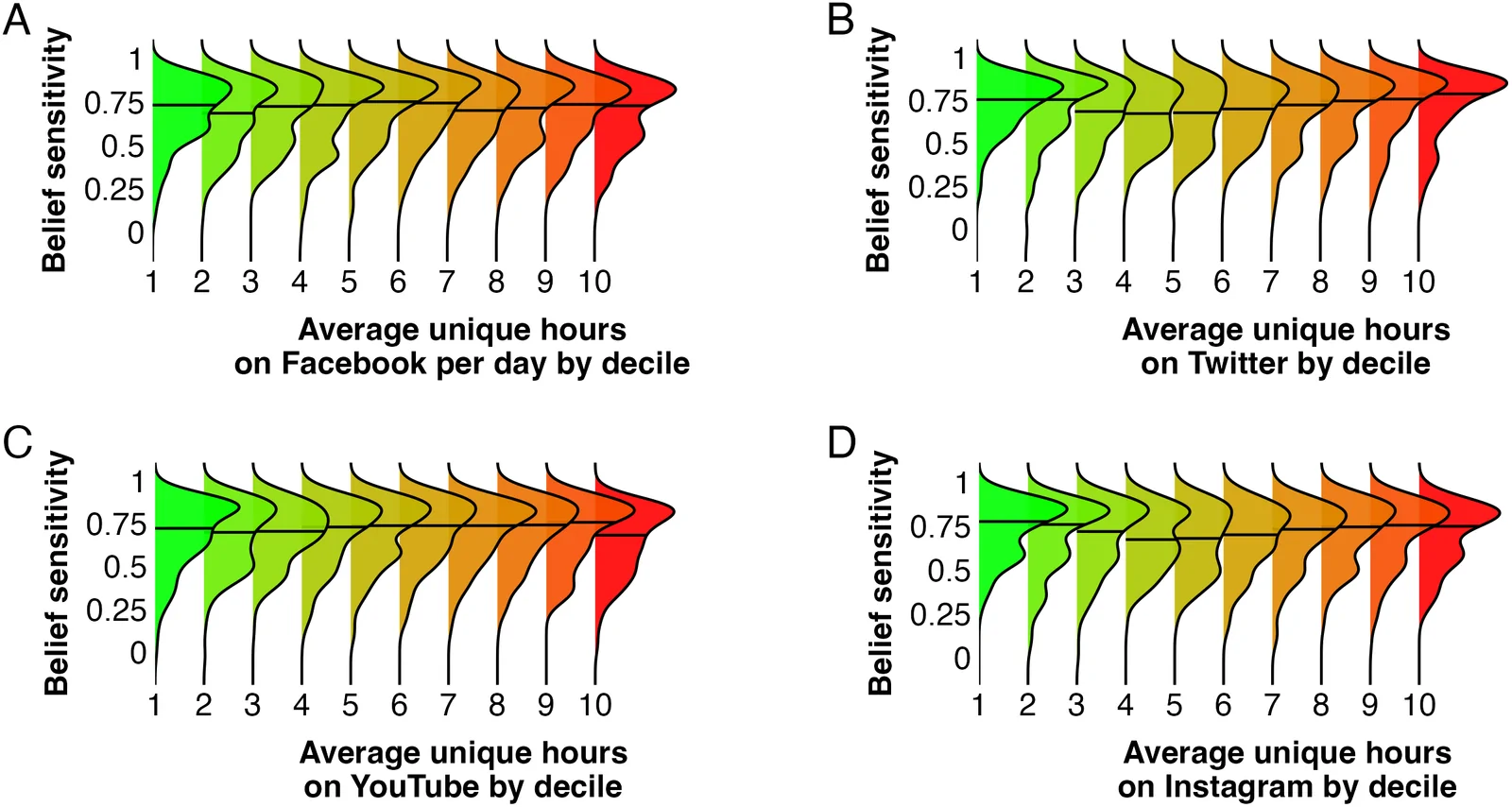
The relation between social media use and belief sensitivity related to COVID-19. Probability density estimates of belief sensitivity for the COVID-19 pandemic by social media use. Panels show the decile of unique hours in the month on (A) Facebook, (B) Twitter, (C) YouTube, and (D) Instagram during the story collection period.

The relations observed above do not consider the panel structure of the data or the fact that participants vary in their individual characteristics in ways that might influence the relations of interest. Further, these simple comparisons do not take advantage of the fact that we have time-dynamic data on both social media use and misperceptions that enable us to further probe their relation.

To account for multiple observations of the same individual and to take advantage of the time-dynamic nature of our data, we used within-between regression models that combine the advantages of fixed-effect regression models that have strong controls for individual-level confounders and random-effects regression models that enable interpretation of individual-level variables. Such models also enable the modeling and interpretation of cross-level interactions to assess whether the effect of social media use (a time-dynamic measure) might be contingent on stable individual differences (a one-time measure).

The possible relation between social media use and belief sensitivity might vary across individual-level characteristics in predictable ways. There are many possible moderators of the relation, but here we examine two factors that have proven important in prior research: whether social media use’s effect on political belief accuracy is moderated by whether a participant indicates that the social media platform (Facebook, Twitter, YouTube, or Instagram) is their most important source of information [18]. The relation between social media use and misperceptions is likely to be strongest among those who describe the platform to be most important. Additional details about these models are available in the Materials and Methods and the supplementary materials in S1 File.

As shown in Fig 4, there is only one significant relation between social media use in the panel on the 2020 U.S. presidential election, however measured, and belief sensitivity. This is the result for self-reported YouTube use (B = 0.011, SE = 0.003, p < 0.001). This result suggests that increased self-reported YouTube use is associated with modestly improved belief sensitivity. The relation is not significant when using digital trace data. In all other cases, whether examining the main effect of social media use or the effect as moderated by social media being an important source, the confidence intervals include zero.

**Fig 4:**
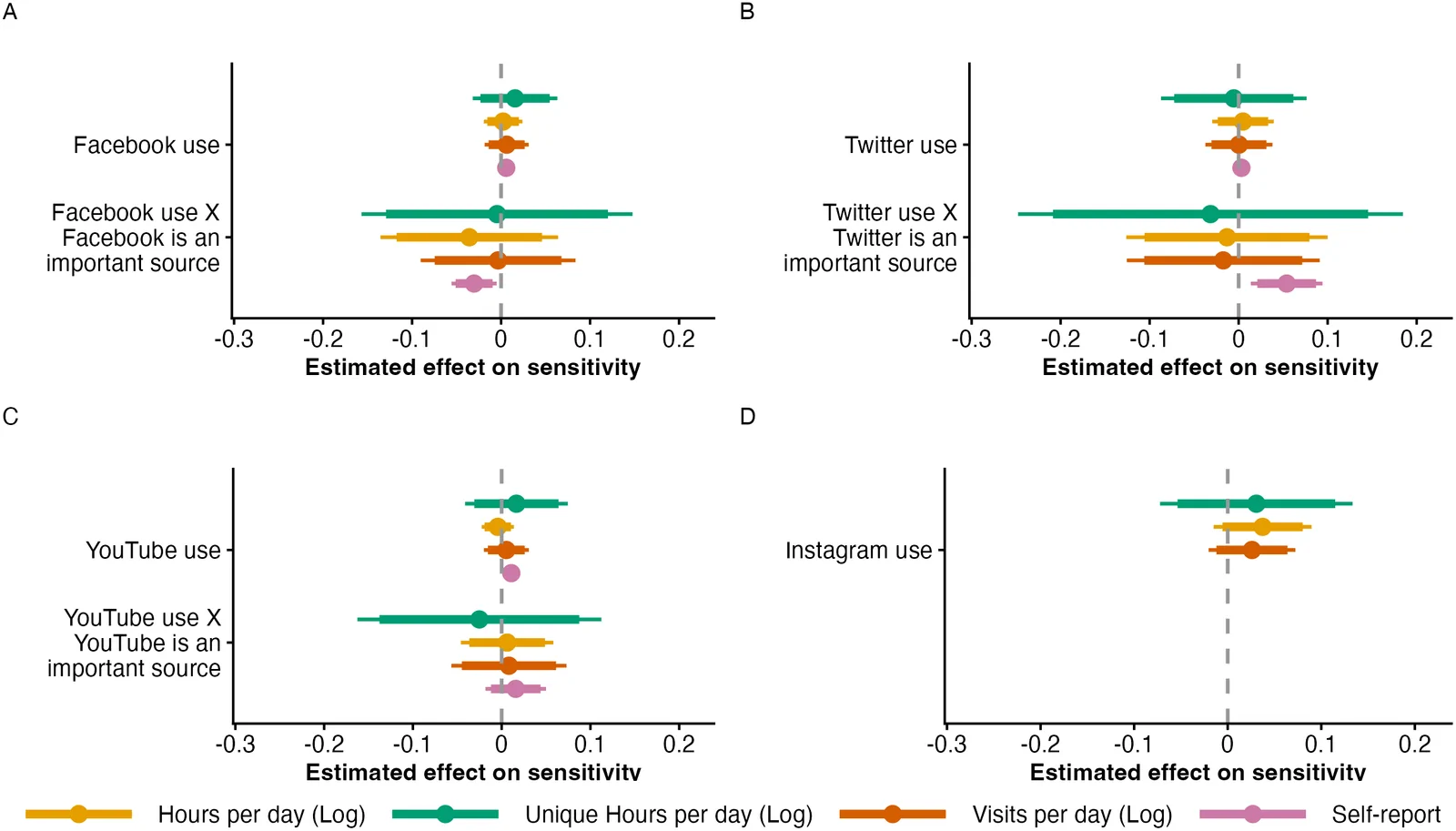
Estimated effects of social media use on belief sensitivity using data from the panel on the 2020 U.S. presidential election. Points represent coefficient estimates from within-between regression models (as described in Materials and Methods). Thick lines represent 95% confidence intervals. Thin lines represent confidence intervals after Bonferroni correction for multiple testing. See supplementary information tables S5-S12 available in S1 File for full details on these models.

A similar pattern is observed in the COVID-19 dataset. As shown in Fig 5, there are two significant relations between social media use and belief sensitivity in this panel. These results are for self-reported YouTube use (B = 0.008, SE = 0.002, p = 0.001) and self-reported Twitter use (B = 0.008, SE = 0.003, p = 0.01), both of which are associated with modestly improved belief sensitivity. Again, however, measures of YouTube and Twitter use that are based on digital trace data show no significant relation with belief sensitivity. In all other cases, whether examining the main effect of social media use or the effect as moderated by social media being an important source, the confidence intervals include zero.

**Fig 5:**
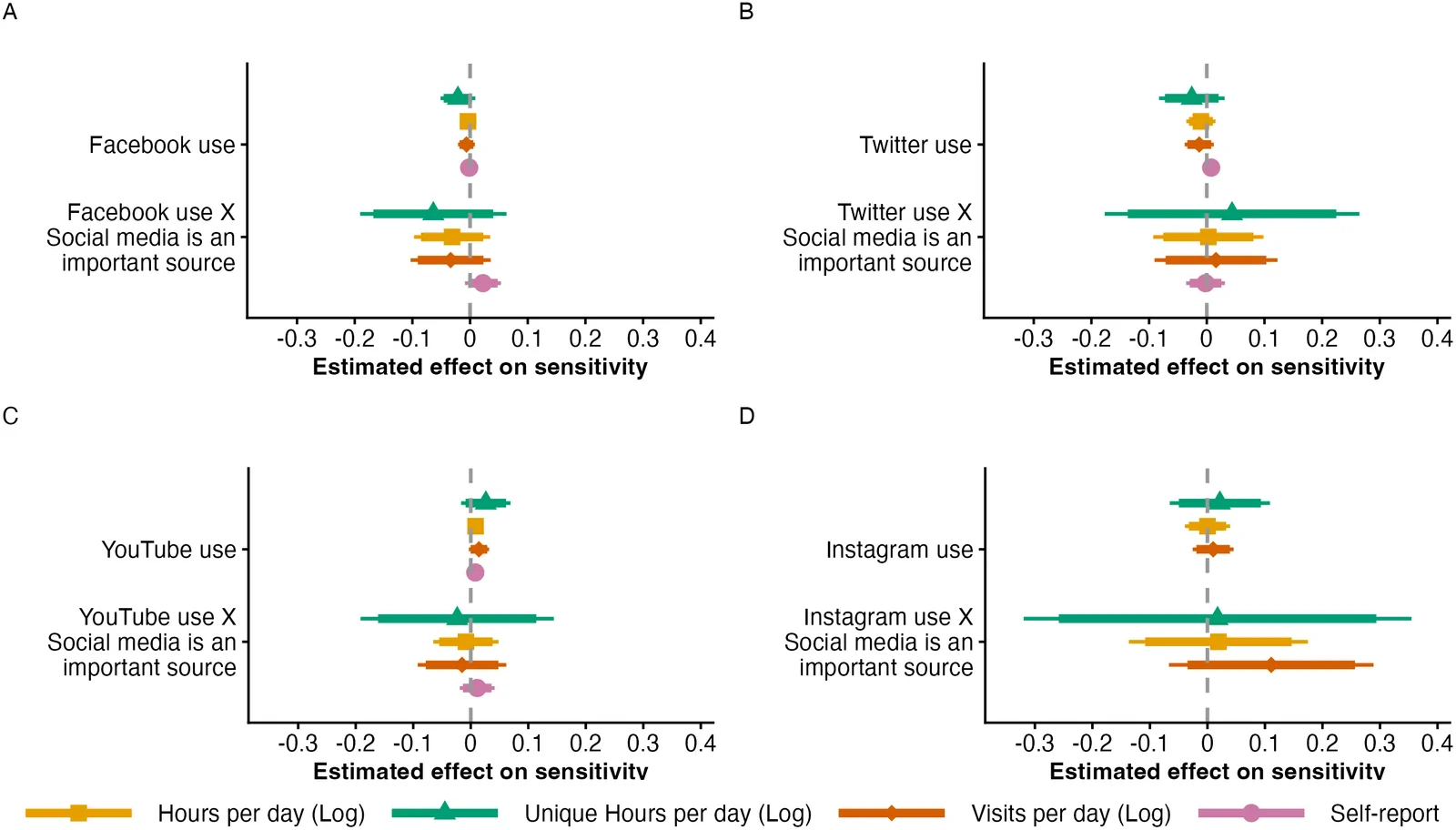
Estimated effects of social media use on belief sensitivity using data from the panel on the COVID-19. Points represent coefficient estimates from within-between regression models (as described in Materials and Methods). Thick lines represent 95% confidence intervals. Thin lines represent confidence intervals after Bonferroni correction for multiple testing. See supplementary information tables S9-S20 available in S1 File for full details on these models.

As a robustness check, we also examined whether the relation between social media use and belief sensitivity was moderated by political ideology. Tables S13-S15 and Tables S24-S26 available in S1 File show full results of these models. Overall, there was no consistent evidence that the relation between social media use and belief sensitivity was moderated by political ideology.

## Discussion

This study provides a robust test of the relation between social media use and Americans’ belief sensitivity, that is, their ability to distinguish between true and false claims. Importantly, the study examines this association in two high-profile domains, politics and public health – domains in which there has been significant scholarly and public concern about the extent to which social media might be responsible for public misperceptions. Our data include participants’ assessments of a wide array of widely circulating claims, both true and false, that people were likely to encounter on social media. We used both self-reported and behavioral measures of social media use and tested for relations among those who say that social media is an important source of information for them. Across all analyses, we see no evidence that social media use has a substantively significant relation with people’s ability to distinguish true and false information. Small but statistically significant positive associations were evident when analyzing self-reported use, but not behavioral measures. Although this could reflect limitations of the behavioral measures (e.g., not logging every device used), we suspect that it is a byproduct of well-known biases in self-reported social media use [28]. Importantly, our analyses include two more visually oriented social media platforms: YouTube and Instagram. Research has shown that misinformation is more prevalent in visual than text-based content but use of such media in this study does not translate into lower belief sensitivity [29].

Given the scale of the data, the longitudinal design, and the use of both self-reported and behavioral measures, the absence of consistent associations places meaningful constraints on claims about the broad effects of social media use on belief sensitivity. In particular, the results are inconsistent with the idea that greater overall exposure to major social media platforms produces large or widespread differences in individuals’ ability to distinguish true from false claims. Rather, the observed patterns align with the “minimal effects” tradition in communication research, which emphasizes that media exposure typically has limited direct effects on mass publics and that observed effects are often contingent on predispositions, contexts, and patterns of use [14].

These findings suggest that social media use had a limited relation to misperceptions in this study, particularly when social media use is measured as time spent on major platforms. However, we do not regard the findings as evidence that social media use never matters for misperceptions. Rather, in the context of the 2020 U.S. presidential election and the COVID-19 pandemic, we find limited evidence that time spent on social media was associated with the misperceptions examined here and, when the two were related, social media use tended to be mildly positively related with ability to distinguish true from false statements.

If there are contexts in which social media use is associated with reduced ability to distinguish between true and false statements, researchers should consider approaches to understanding such patterns. One might have to consider whether measurement of social media use based on time spent on a platform might obscure meaningful differences in how Americans actually use these platforms. While focusing on the overall volume of platform use provides a measure of the extent to which respondents allocate time to social media, it does not distinguish among different activities (e.g., posting, sharing, commenting, messaging, or passive browsing), exposure to specific false or misleading claims, the credibility of sources encountered, algorithmic curation, interpersonal discussion, engagement with particular kinds of political and health-related content, or the composition of user’s networks. As a result, it might be that discovering any relation between social media use and reduced ability to distinguish between true and false claims might require more granular attention to variations in how people engage with platforms and the types of information they encounter. More generally, associations between social media use and misperceptions might vary across platforms, time periods, political contexts, and subpopulations, or might be concentrated among specific forms of use that are obscured when use is measured in aggregate.

The study has important limitations. Our evidence is consistent with the idea that social media use does not have large, broad effects on susceptibility to widely circulating false stories across all individuals. This does not, however, mean that social media does not promote misperceptions among some people or in domains other than those examined here. It is possible that for certain sub-populations social media use is harmful to their ability to distinguish true and false information. It is also possible that platforms with more limited content moderation policies, such as the video sharing platform Rumble or Truth Social, might affect users’ susceptibility in a different manner than the platforms included in this study. Further research into how social media use does or does not affect particular groups would be valuable. Prior work has shown that a small number of people consume a disproportionate amount of false information circulating online [30]. It is possible that the relations we observe would be different among this group of people.

Finding similar results across the domains of political campaigns and elections and of public health during the COVID-19 pandemic would seem to be strengths of the study as there is ample evidence of widely circulating false claims in each domain. However, these domains also received substantial public attention, including particularly strong warnings from journalists and others about the threat posed by false or dubious information. Relations between social media use and misperceptions might be different in information environments where there is less public scrutiny or in which fact-checking and efforts to correct false information by news organizations are less robust. Finally, we note that this research was conducted in the United States; the associations discussed here might not be the same in other countries and cultures.

The results reported here do not mean that social media use is unrelated to the contemporary prevalence of misinformation in the U.S. information landscape. However, like other recent critiques of the discourse about social media and misinformation, they call into doubt the simplest version of the relation [31]. Spending time using popular social media platforms is not directly related to Americans’ ability to discriminate between truths and falsehoods, even among those who characterize a platform as their most important source of news. Perhaps such effects are limited to small networks of users, particular styles of use, and/or niche platforms with more limited content moderation. Or perhaps the prevalence of misperceptions reflects larger social forces unrelated to social media. These are questions to which scholars must turn next.

## Supporting information

S1 File Supplementary appendix. (PDF)
