# Correction: The limited effects of social media use on misperceptions Social media’s limited role in misperceptions

**DOI:** 10.1371/journal.pone.0362477

**Published:** 2026-11-09

**Authors:** Qin Li, Robert M. Bond, Erik Nisbet, Graham Dixon, Shelly Hovick, Kilhoe Na, Duane Wegener, R. Kelly Garrett

The phrase “Social media’s limited role in misperceptions” was incorrectly included in the title. The correct title is: The limited effects of social media use on misperceptions. The correct citation is: Li Q, Bond RM, Nisbet E, Dixon G, Hovick S, Na K, et al. (2026) The limited effects of social media use on misperceptions. PLoS One 21(9): e0354486. https://doi.org/10.1371/journal.pone.0354486.
