# Supplementary material for: The limited effects of social media use on misperceptions Social media’s limited role in misperceptions
Source: PLoS One. 2026 Sep 30;21(9):e0354486. doi: 10.1371/journal.pone.0354486 (PMC13626341; doi:10.1371/journal.pone.0354486)
Supplement: Supplementary file 1 [file pone.0354486.s001.pdf]

Supplementary Appendix

Details on study design

Panel design

YouGov conducted two separate panels. The first panel covered the 2020 U.S. presidential campaign, with data collection spanning September 17, 2020 to November 23, 2020. Initially, we planned to collect five waves of data, spanning the fall campaign with a final wave occurring after the election. Due to the unprecedented circumstances of the post-election period in 2020, we contracted with YouGov to collect three additional waves, with a refresh of new participants. However, here we only use the originally planned initial five waves. These waves were spaced approximately two weeks apart. YouGov used a sample matching approach for half the sample, to ensure that this was a representative sample (n = 700 at baseline); the sample for which we had behavioral data was a non-probability sample (n = 700 at baseline), and included only people who volunteered to have their web behavior tracked.

The second panel covered the COVID-19 pandemic from December 9, 2020 to June 16, 2021. We collected a monthly panel, with a total of seven waves. The sample was again evenly split between representative and non-probability samples (N = 1200 at baseline).

We used a similar approach to prior work (1) to generate belief statements for both panels. Specifically, we retrieved social media engagement data for news stories that had been shared in the days leading into each panel using the NewsWhip API. For the election panel, we collected the most highly engaged with stories on social media in the preceding 7 days, due to the panel going into the field every two weeks. The rapidly changing election news environment afforded many stories that were verifiably true or false during each week. We used an aggregate number of social media engagement metrics, including reactions, shares, and comments associated with each news story to identify the stories that

had the highest engagement. For the COVID-19 panel, we collected the mostly highly engaged with stories on social media in the preceding month, due to the panel going into the field each month.

Consistent with prior work, for both panels we established story veracity using several indicators, including source domain, news coverage of the story by other outlets, assessments by fact checking organizations, and consultation with expert scholars. The team then drafted short statements ("belief statements") that could be placed on each panel that summarized key claims in the stories. We went to substantial effort to ensure that the statements on the surveys were verifiably true or false given the best available information available at the time that the survey went into the field.

On each panel survey, we asked participants to indicate their own assessment of the veracity of the belief statements on a six- point scale, anchored by "definitely true" and "definitely false." Participants were asked to give their assessment of each statement using their existing knowledge without conducting additional research. It is important to acknowledge that participants' responses, like all survey measures we are aware of, measure how participants portray their beliefs rather than their true beliefs. Participants may, for example, respond strategically in ways that are inconsistent with their true beliefs, perhaps to express support for an idea or person. However, most studies show that expressive responding of this type is infrequent. Further, if participants were to be willing to express these beliefs to others as they are on these surveys, even if they themselves do not actually hold these beliefs, this expression may make the information environment difficult to navigate.

Using participants' assessments of the belief statements, we constructed a measure of their ability to distinguish true and false information using signal detection theory (2). This measure, sensitivity, rates participants ability to identify true and false belief statements correctly and confidently.

To measure social media use we rely on two strategies. First, we included measures of social media use for common platforms on each wave of both panel surveys. For the election panel, we asked participants how frequently they used Facebook, Twitter, and YouTube with a five-point response scale

(5 = “several times a day”, 4 = “about once a day”, 3 = “a few times a week”, 2 = “every few weeks”, and 1 = “less often”). For the COVID-19 panel, we asked participants how frequently they had visited Facebook, Twitter, and YouTube in the prior week on a similar five-point response scale (5 = “several times a day”, 4 = “once”, 3 = “a few times”, 2 = “once”, and 1 = “Never/Not on [platform]”).

For both panels, we also contracted with YouGov to provide log data on participants use of apps and the web during the course of the panel. For the election panel, we contracted to have half of the panel provide this data, and for the COVID-19 panel we contracted for the full panel to provide it. For both the election panel (N = 624) and the COVID-19 panel (N = 1010), most but not all participants who had their app and web usage tracked provided sufficient data to construct social media use measures using their tracked data.

Question wording

We included self-reported measures of social media use for common platforms on each wave of both panel surveys. For the election panel, we asked participants how frequently they used Facebook, Twitter, and YouTube with a five-point response scale (5 = “several times a day”, 4 = “about once a day”, 3 = “a few times a week”, 2 = “every few weeks”, and 1 = “less often”). For the COVID-19 panel, we asked participants how frequently they had visited Facebook, Twitter, and YouTube in the prior week on a similar five-point response scale (5 = “several times a day”, 4 = “once”, 3 = “a few times”, 2 = “once”, and 1 = “Never/Not on [platform]”).

We also included measures of the importance of the social media platforms as a source of news. In the election panel, we asked participants which of a list of sources they considered to be their most important source of news. Among the listed options, participants could choose Facebook (N = 113), Twitter (N = 46), and YouTube (N = 61). Using this variable, we created three indicator variables for whether participants considered each platform an important source. In the COVID-19 panel, we asked a

similar question about what their most important source of information about the COVID-19 pandemic was. Among the response options was "Information found on social media platforms (Facebook, Twitter, Instagram, etc.)." Using this variable, we created a single indicator variable for whether a participant considered social media, in general, an important source.

Belief statements

Table S1 shows example belief statements from the panel on the 2020 U.S. presidential election. Table S2 shows example belief statements from the panel on COVID-19.

Behavioral data

Details on behavioral data collection

To be qualified for the YouGov Pulse panel, each panelist is required to have a Windows, Mac OS and/or Android device. Pulse participants must transmit a minimum of 15 days of data per month to remain on the YouGov Pulse panel. Pulse participants do not have to take specific actions for transmission to occur (transmissions occur automatically while the YouGov Pulse app remains active on the participant's device). All rules for joining and remaining in the YouGov Pulse panel are explained to the participant at sign up.

Participants are informed at sign up about which data YouGov Pulse collects. YouGov Pulse participants are able to stop data collection by uninstalling the YouGov Pulse app and browser extensions (and are given specific instructions for doing so for different operating systems). If the user only removes the app or browser extensions, their data are not automatically deleted from the data set. However, specific requests to delete data can be sent by panelists to YouGov. In such cases, YouGov will delete all historical YouGov Pulse data for that respondent.

We capture domain-level data for any browser where the browser extension is installed, and we capture both domain and sub-domain level data. YouGov Pulse captures data from most commonly used browsers. The full list of supported browsers consists of Chrome, FireFox, Safari, Microsoft Edge, and the Huawei and Samsung browsers. As long as the application is installed and extensions are enabled, YouGov Pulse will capture browser data. When the user uses a browser on their smartphone or tablet, with Pulse installed, YouGov Pulse will collect domain-level data. The constraints are only on the browsers from which data can be collected, as indicated above.

Data capture is paused while the respondent is in privacy mode. Episodes of privacy mode use are not captured by YouGov Pulse. Such episodes would simply result in gaps in data capture.

Recruitment language

The following text was used to recruit participants to the surveys:

Earn $50 cash this year just for browsing the internet!

YouGov is partnering with Facebook to understand how people use the internet and social networks.

We are interested in which web sites you visit, which ads you see, which apps you use, and your social media activity. To participate in YouGov Pulse you must install an app on a Windows, Mac OS and/or Android device. YouGov Pulse is not currently available on iOS. You will receive 3000 points each month in which you transmit for a minimum number of days. After transmitting for 12 months, you will receive a bonus 14,000 points (a total of 50,000 points) and you will be eligible to redeem your 50,000 points for $50 cash!

Requirements to participate: Windows, Mac OS and/or Android platforms. (not available on iOS).

Behavioral data

Pre-processing

We used three different datasets from log behavioral data to create measures. They include the web dataset, which consists of URL-level web visits on desktops and tablets, the app dataset, which records App-level visits on mobile phones, and the media dataset, recording URL-level visits to video-based media (e.g., YouTube).

We processed the data using the following procedure. We only kept URL-level and App-level visits to Facebook, Twitter, YouTube, and Instagram—four social media platforms our panelists used the most. We then inspected the anonymized URLs in the URL-level data. Specifically, we examined whether each visit accurately captures social media activities for ordinary users (as opposed to social media use for, e.g., developers). We also examined whether using the anonymized URLs enabled us to distinguish between different types of social media visits to approximate social media use pertaining to information consumption. Throughout this process, we observed that participants could spend their time on various activities (e.g., video games, messages, video calls, marketplace) and miscellaneous functions (e.g., settings, logins, and authentications) that may or may not be related to exposure to (mis)information, leading to slight overestimation of visits to social media for information. After evaluating the extent to which each type of social media visit is likely to be related to exposure to (mis)information and assess whether they can be consistently identified across different devices, we decided to remove visits to Facebook video games on all devices while retaining others. A more detailed explanation for our criteria can be found below.

Criteria and decisions for including/excluding specific types of visits: 1. There is no reason to expect that playing games on social media will be related to exposure to (mis)information about politics

and health with any frequency. Therefore, we exclude social media-based gaming from our estimates of social media use in both the web and app data. 2. Private messages sent via a social media service (versus posts) can be related to exposure to (mis)information (e.g., people can receive links to articles through private messages). Therefore, we include these activities in our estimates of social media use. 3. Miscellaneous functions such as settings, logins, and authentications can be related to exposure to (misinformation). Some individuals may deliberatively customize privacy settings or sign out their accounts to view low-credibility content. However, miscellaneous functions do not constitute information exposure, and therefore, it simply adds some noise to the measures but should not systematically bias our estimates. While our goal is to minimize measurement errors as much as possible, removing these visits on all devices is also infeasible given the limitations of the dataset.

Details on samples

Sample characteristics

Table S3 shows demographic information from the panel on the 2020 U.S. presidential election. Table S4 shows demographic information from the panel on COVID-19.

Details on social media use measures

Using the logged data, we constructed three separate, but interrelated measures of social media use. First, we tracked the average total duration of time that a participant spent on each of Facebook, Twitter, YouTube and Instagram per day during the period that highly-engaged with stories were observed for each panel. That is, we took the average of participants social media use per day for each platform for the week during which election-related stories were collected from NewsWhip for the election panel and the average social media use per day for each platform for the month during which Covid-19-related

stories were collected from NewsWhip for the Covid-19 panel. Second, we measured the average total number of visits per day that a participant made to each of the same four social media platforms during the story observation period. Finally, we created a third measure that was the number of average number of unique hours per day that a participant visited a social media platform. That is, if a participant visited a given platform during the 1:00 – 1:59 hour, regardless of the duration of the visit or the number of visits logged, we would count them as having visited during that hour. We then summed the total number of hours during which a participant visited a platform at least once and measured the average total number of unique hours per day.

Analytic approach

We estimated the relationship between social media use and belief sensitivity using a series of within-between regression models (3). Within-between models enable researchers to combine the benefits of fixed-effects regression, with a high degree of control for differences across participants, with random-effects regression that enables for the observation of between-participant differences. Further, this estimation strategy enables cross-level interactions between time-variant variables that are observed in each wave and time-invariant variables that are stable across waves.

For all models, we present both the 95% confidence intervals from the original models and the Bonferroni-corrected confidence intervals that account for the fact that we are testing similar relationships across these models. In nearly all cases aside from the significant relationships for self-reported social media use noted in the main text, the confidence intervals for the estimated effects of social media use on misperceptions include zero regardless of which confidence intervals are used.

Regression results

Table S5 shows the results of within-between regression models of sensitivity with time-variant behavioral social media use as measured using the *average total duration of time* a participant visited a given social media platform as predictors for the panel covering the 2020 U.S. presidential election.

Table S6 shows the results of within-between regression models of sensitivity with time-variant behavioral social media use as measured using the average total duration of time a participant visited a given social media platform, including *interactions* between social media use and whether a participant considers social media an important source, as predictors for the panel covering the 2020 U.S. presidential election.

Table S7 shows the results of within-between regression models of sensitivity with time-variant behavioral social media use as measured using the *average number of unique hours* a participant visited a given social media platform as predictors for the panel covering the 2020 U.S. presidential election.

Table S8 shows the results of within-between regression models of sensitivity with time-variant behavioral social media use as measured using the average number of unique hours a participant visited a given social media platform, including *interactions* between social media use and whether a participant considers social media an important source, as predictors for the panel covering the 2020 U.S. presidential election.

Table S9 shows the results of within-between regression models of sensitivity with time-variant behavioral social media use as measured using the *average total number of times* a participant visited a given social media platform as predictors for the panel covering the 2020 U.S. presidential election.

Table S10 shows the results of within-between regression models of sensitivity with time-variant behavioral social media use as measured using the average total number of times a participant visited a given social media platform, including *interactions* between social media use and whether a partic-

ipant considers social media an important source, as predictors for the panel covering the 2020 U.S. presidential election.

Table S11 shows the results of within-between regression models of sensitivity with time-variant behavioral social media use as measured using *self-reported social media use* on a given social media platform as predictors for the panel covering the 2020 U.S. presidential election.

Table S12 shows the results of within-between regression models of sensitivity with time-variant behavioral social media use as measured using self-reported social media use on a given social media platform, including *interactions* between social media use and whether a participant considers social media an important source, as predictors for the panel covering the 2020 U.S. presidential election.

Table S13 shows the results of within-between regression models of belief sensitivity with time-variant behavioral social media use as measured using average time spent on social media, including *interactions* between social media use and political ideology for the panel covering the 2024 U.S. election.

Table S14 shows the results of within-between regression models of belief sensitivity with time-variant behavioral social media use as measured using average unique hours spent on social media, including *interactions* between social media use and political ideology for the panel covering the 2024 U.S. election.

Table S15 shows the results of within-between regression models of belief sensitivity with time-variant behavioral social media use as measured using average total number of times a participant visited a given social media platform, including *interactions* between social media use and political ideology for the panel covering the 2024 U.S. election.

Table S16 shows the results of within-between regression models of sensitivity with time-variant behavioral social media use as measured using the *average total duration of time* a participant visited a given social media platform as predictors for the panel covering the *COVID-19 pandemic*.

Table S17 shows the results of within-between regression models of sensitivity with time-variant behavioral social media use as measured using the average total duration of time a participant visited a given social media platform, including *interactions* between social media use and whether a participant considers social media an important source, as predictors for the panel covering the COVID-19 pandemic.

Table S18 shows the results of within-between regression models of sensitivity with time-variant behavioral social media use as measured using the *average number of unique hours* a participant visited a given social media platform as predictors for the panel covering the COVID-19 pandemic.

Table S19 shows the results of within-between regression models of sensitivity with time-variant behavioral social media use as measured using the average number of unique hours a participant visited a given social media platform, including *interactions* between social media use and whether a participant considers social media an important source, as predictors for the panel covering the COVID-19 pandemic.

Table S20 shows the results of within-between regression models of sensitivity with time-variant behavioral social media use as measured using the *average total number of times* a participant visited a given social media platform as predictors for the panel covering the COVID-19 pandemic.

Table S21 shows the results of within-between regression models of sensitivity with time-variant behavioral social media use as measured using the average total number of times a participant visited a given social media platform, including *interactions* between social media use and whether a participant considers social media an important source, as predictors for the panel covering the COVID-19 pandemic.

Table S22 shows the results of within-between regression models of sensitivity with time-variant behavioral social media use as measured using *self-reported social media use* on a given social media platform as predictors for the panel covering the COVID-19 pandemic.

Table S23 shows the results of within-between regression models of sensitivity with time-variant behavioral social media use as measured using self-reported social media use on a given social media platform, including *interactions* between social media use and whether a participant considers social media an important source, as predictors for the panel covering the COVID-19 pandemic.

Table S24 shows the results of within-between regression models of belief sensitivity with time-variant behavioral social media use as measured using average time spent on social media, including *interactions* between social media use and political ideology for the panel covering the COVID-19 pandemic.

Table S25 shows the results of within-between regression models of belief sensitivity with time-variant behavioral social media use as measured using average unique hours spent on social media, including *interactions* between social media use and political ideology for the panel covering the COVID-19 pandemic.

Table S26 shows the results of within-between regression models of belief sensitivity with time-variant behavioral social media use as measured using average total number of times a participant visited a given social media platform, including *interactions* between social media use and political ideology for the panel covering the COVID-19 pandemic.

Wave	Veracity	Statement
1	False	Vice Presidential Candidate Kamala Harris has endorsed a California state senator who proposed legalizing pedophilia if the age difference between the adult and minor is less than 10 years.
1	True	At least four boats sank and several more required emergency assistance during a boat parade in support of President Trump held on Lake Travis near Austin, Texas.
3	False	The Washington Post sent a tweet encouraging readers to imagine a world without President Trump in response to the President's COVID-19 diagnosis.
3	True	President Trump and first lady Melania Trump tested positive for COVID-19.
5	False	A flaw in election software used to tabulate ballots throughout Michigan caused officials in Antrim County to inaccurately declare Joe Biden the winner before detecting the programming error.
5	True	A woman who says she was fired from her job in an Ohio sherriff's office for being gay has been elected sherriff, replacing the man who fired her.

Table S1: Example belief statements from the panel on the 2020 U.S. presidential election.

2

Wave	Veracity	Statement
1	False	The Governor of Pennsylvania announced a new law requiring individuals living in the same household to wear masks while inside their own home.
1	True	Early-stage Phase III trial results suggest that Pfizer's COVID-19 vaccine is more than 90% effective at preventing coronavirus infection.
3	False	The Biden Administration lost 20 million doses of COVID-19 vaccine that were shipped to states under its new distribution plan.
3	True	The CDC recommends that people continue to wear masks and socially distance after getting the COVID-19 vaccine.
5	False	Less than 10% of the funding in the recent $1.9 trillion COVID-19 relief bill passed by Congress was related to the pandemic.
5	True	Country singer Dolly Parton, who helped fund the development of the Moderna COVID-19 vaccine, received her vaccination on camera.

Table S2: Example belief statements from the panel on COVID-19.

	Number	Percent
N	1400	100.0
Age		
18-29	179	12.7
30-44	379	27.1
45-59	370	26.4
60+	472	33.7
Gender		
Female	754	53.9
Race		
White	938	67.0
Black	173	12.4
Hispanic	199	14.2
Asian	41	2.9
Native American	14	1.0
Two or more races	28	2.0
Education		
No high school	38	2.7
High school graduate	406	29.0
Some college	311	22.2
2-year degree	177	12.6
4-year degree	296	21.1
Post-graduate degree	172	12.3
Political ideology		
Very liberal	203	14.5
Liberal	197	14.1
Somewhat liberal	115	8.2
Moderate	437	31.2
Somewhat conservative	110	7.9
Conservative	186	13.3
Very conservative	157	11.2

Table S3: Sample demographics from the panel on the 2020 U.S. presidential election.

	Number	Percent
N	1200	100.0
Age		
18-29	113	9.4
30-44	302	25.2
45-69	321	26.8
60+	464	38.7
Gender		
Female	693	57.8
Race		
White	921	76.8
Black	107	8.9
Asian	42	3.5
Education		
No high school	29	2.4
High school graduate	291	24.3
Some college	285	23.8
2-year degree	161	13.4
4-year degree	274	22.8
Post-graduate degree	160	13.3
Political ideology		
Very liberal	182	15.2
Liberal	176	14.7
Somewhat liberal	101	8.4
Moderate	381	31.8
Somewhat conservative	100	8.3
Conservative	121	10.1
Very conservative	139	11.6

Table S4: Sample demographics from the panel on COVID-19.

	Election panel, DV: Sensitivity (AUC)
	Main effects (no interactions)
Within-participant effects	
Duration on Facebook$_t$	0.002 (0.009)
Duration on Twitter$_t$	0.005 (0.014)
Duration on YouTube$_t$	−0.004 (0.008)
Duration on Instagram$_t$	0.037 (0.022)
Between-participant effects	
Duration on Facebook (mean$_i$)	0.003 (0.009)
Duration on Twitter (mean$_i$)	0.024 (0.017)
Duration on YouTube (mean$_i$)	−0.005 (0.010)
Duration on Instagram (mean$_i$)	−0.014 (0.022)
Age	0.001 (0.000)**
Female	0.004 (0.012)
Education	0.013 (0.004)**
Ideology (conservatism high)	−0.063 (0.003)***
News interest	0.034 (0.007)***
Facebook an important source	−0.071 (0.025)**
Twitter an important source	0.006 (0.045)
Youtube an important source	−0.066 (0.028)*
(Intercept)	0.721 (0.037)***
AIC	−2794.365
BIC	−2662.171
Log Likelihood	1420.183
Num. obs.	2316
Num. groups: anon_id	487
Var: anon_id (Intercept)	0.013
Var: Residual	0.011

$^{***}p < 0.001$; $^{**}p < 0.01$; $^{*}p < 0.05$

Table S5: Results of regression with sensitivity in wave$_t$ as the outcome variable using the panel on the 2020 U.S. election. Variables with t subscript are measured in each time point. Within-participant effects are difference in difference estimates of within-participant changes from time-variant variables. Between-participant effects are estimates of differences between participants from time-invariant variables.

	Election panel, DV: Sensitivity (AUC)		
	Facebook interaction	Twitter interaction	YouTube interaction
Cross-level interactions			
Duration on Facebook$_t$ × Facebook an important source	−0.036 (0.042)		
Duration on Twitter$_t$ × Twitter an important source		−0.013 (0.047)	
Duration on YouTube$_t$ × YouTube an important source			0.006 (0.022)
Within-participant effects			
Duration on Facebook$_t$	0.004 (0.009)	0.002 (0.009)	0.002 (0.009)
Duration on Twitter$_t$	0.004 (0.014)	0.006 (0.015)	0.005 (0.014)
Duration on YouTube$_t$	−0.004 (0.008)	−0.004 (0.008)	−0.005 (0.008)
Duration on Instagram$_t$	0.037 (0.022)	0.037 (0.022)	0.036 (0.022)
Between-participant effects			
Duration on Facebook (mean$_i$)	0.003 (0.009)	0.003 (0.009)	0.003 (0.009)
Duration on Twitter (mean$_i$)	0.024 (0.017)	0.024 (0.017)	0.024 (0.017)
Duration on YouTube (mean$_i$)	−0.005 (0.010)	−0.005 (0.010)	−0.005 (0.010)
Duration on Instagram (mean$_i$)	−0.014 (0.022)	−0.014 (0.022)	−0.014 (0.022)
Age	0.001 (0.000)**	0.001 (0.000)**	0.001 (0.000)**
Female	0.004 (0.012)	0.004 (0.012)	0.004 (0.012)
Education	0.013 (0.004)**	0.013 (0.004)**	0.013 (0.004)**
Ideology (conservatism high)	−0.063 (0.003)***	−0.063 (0.003)***	−0.063 (0.003)***
News interest	0.034 (0.007)***	0.034 (0.007)***	0.034 (0.007)***
Facebook an important source	−0.071 (0.025)**	−0.071 (0.025)**	−0.071 (0.025)**
Twitter an important source	0.006 (0.045)	0.006 (0.045)	0.006 (0.045)
Youtube an important source	−0.066 (0.028)*	−0.066 (0.028)*	−0.067 (0.028)*
(Intercept)	0.721 (0.037)***	0.721 (0.037)***	0.721 (0.037)***
AIC	−2788.582	−2788.176	−2786.629
BIC	−2650.639	−2650.233	−2648.687
Log Likelihood	1418.291	1418.088	1417.315
Num. obs.	2316	2316	2316
Num. groups: anon_id	487	487	487
Var: anon_id (Intercept)	0.013	0.013	0.013
Var: Residual	0.011	0.011	0.011

$^{***}p < 0.001$; $^{**}p < 0.01$; $^{*}p < 0.05$

Table S6: Results of regression with sensitivity in wave$_t$ as the outcome variable using the panel on the 2020 U.S. election. Variables with t subscript are measured in each time point. Cross-level interactions are interactions between time-variant and time-invariant variables. Within-participant effects are difference in difference estimates of within-participant changes from time-variant variables. Between-participant effects are estimates of differences between participants from time-invariant variables.

	Election panel, DV: Sensitivity (AUC)
	Main effects (no interactions)
Within-participant effects	
Unique hours on Facebook_t	0.016 (0.020)
Unique hours on Twitter_t	−0.005 (0.034)
Unique hours on YouTube_t	0.017 (0.024)
Unique hours on Instagram_t	0.031 (0.043)
Between-participant effects	
Unique hours on Facebook (mean_i)	0.020 (0.018)
Unique hours on Twitter (mean_i)	0.034 (0.032)
Unique hours on YouTube (mean_i)	−0.028 (0.026)
Unique hours on Instagram (mean_i)	−0.034 (0.042)
Age	0.001 (0.000)*
Female	0.002 (0.012)
Education	0.014 (0.004)**
Ideology (conservatism high)	−0.063 (0.003)***
News interest	0.034 (0.007)***
Facebook an important source	−0.073 (0.025)**
Twitter an important source	0.016 (0.046)
Youtube an important source	−0.063 (0.028)*
(Intercept)	0.723 (0.036)***
AIC	−2807.447
BIC	−2675.252
Log Likelihood	1426.723
Num. obs.	2316
Num. groups: anon_id	487
Var: anon_id (Intercept)	0.013
Var: Residual	0.011

$^{***}p < 0.001$; $^{**}p < 0.01$; $^{*}p < 0.05$

Table S7: Results of regression with sensitivity in wave_t as the outcome variable using the panel on the 2020 U.S. election. Variables with t subscript are measured in each time point. Within-participant effects are difference in difference estimates of within-participant changes from time-variant variables. Between-participant effects are estimates of differences between participants from time-invariant variables.

	Election panel, DV: Sensitivity (AUC)		
	Facebook interaction	Twitter interaction	YouTube interaction
Cross-level interactions			
Unique hours on Facebook$_t$ × Facebook an important source	−0.005 (0.064)		
Unique hours on Twitter$_t$ × Twitter an important source		−0.032 (0.090)	
Unique hours on YouTube$_t$ × YouTube an important source			−0.025 (0.057)
Within-participant effects			
Unique hours on Facebook$_t$	0.016 (0.020)	0.016 (0.020)	0.016 (0.020)
Unique hours on Twitter$_t$	−0.006 (0.034)	−0.001 (0.036)	−0.006 (0.034)
Unique hours on YouTube$_t$	0.017 (0.024)	0.017 (0.024)	0.020 (0.025)
Unique hours on Instagram$_t$	0.031 (0.043)	0.029 (0.043)	0.034 (0.044)
Between-participant effects			
Unique hours on Facebook (mean$_i$)	0.020 (0.018)	0.020 (0.018)	0.020 (0.018)
Unique hours on Twitter (mean$_i$)	0.034 (0.032)	0.034 (0.032)	0.034 (0.032)
Unique hours on YouTube (mean$_i$)	−0.028 (0.026)	−0.028 (0.026)	−0.028 (0.026)
Unique hours on Instagram (mean$_i$)	−0.034 (0.042)	−0.034 (0.042)	−0.034 (0.042)
Age	0.001 (0.000)*	0.001 (0.000)*	0.001 (0.000)*
Female	0.002 (0.012)	0.002 (0.012)	0.002 (0.012)
Education	0.014 (0.004)**	0.014 (0.004)**	0.014 (0.004)**
Ideology (conservatism high)	−0.063 (0.003)***	−0.063 (0.003)***	−0.063 (0.003)***
News interest	0.034 (0.007)***	0.034 (0.007)***	0.034 (0.007)***
Facebook an important source	−0.073 (0.025)**	−0.073 (0.025)**	−0.073 (0.025)**
Twitter an important source	0.016 (0.046)	0.016 (0.046)	0.016 (0.046)
Youtube an important source	−0.063 (0.028)*	−0.063 (0.028)*	−0.063 (0.028)*
(Intercept)	0.723 (0.036)***	0.723 (0.036)***	0.723 (0.036)***
AIC	−2801.779	−2802.598	−2801.761
BIC	−2663.837	−2664.656	−2663.818
Log Likelihood	1424.889	1425.299	1424.880
Num. obs.	2316	2316	2316
Num. groups: anon_id	487	487	487
Var: anon_id (Intercept)	0.013	0.013	0.013
Var: Residual	0.011	0.011	0.011

$^{***}p < 0.001$; $^{**}p < 0.01$; $^{*}p < 0.05$

Table S8: Results of regression with sensitivity in wave$_t$ as the outcome variable using the panel on the 2020 U.S. election. Variables with t subscript are measured in each time point. Cross-level interactions are interactions between time-variant and time-invariant variables. Within-participant effects are difference in difference estimates of within-participant changes from time-variant variables. Between-participant effects are estimates of differences between participants from time-invariant variables.

	Election panel, DV: Sensitivity (AUC)
	Main effects (no interactions)
Within-participant effects	
Mean visits on Facebook_t	0.006 (0.010)
Mean visits on Twitter_t	0.000 (0.016)
Mean visits on YouTube_t	0.005 (0.011)
Mean visits on Instagram_t	0.026 (0.019)
Between-participant effects	
Mean visits on Facebook (mean_i)	0.005 (0.010)
Mean visits on Twitter (mean_i)	0.018 (0.016)
Mean visits on YouTube (mean_i)	−0.017 (0.014)
Mean visits on Instagram (mean_i)	0.005 (0.025)
Age	0.001 (0.000)**
Female	0.003 (0.012)
Education	0.013 (0.004)**
Ideology (conservatism high)	−0.064 (0.003)***
News interest	0.034 (0.007)***
Facebook an important source	−0.073 (0.025)**
Twitter an important source	0.017 (0.044)
Youtube an important source	−0.065 (0.029)*
(Intercept)	0.725 (0.037)***
AIC	−2796.485
BIC	−2664.291
Log Likelihood	1421.243
Num. obs.	2316
Num. groups: anon_id	487
Var: anon_id (Intercept)	0.013
Var: Residual	0.011

***$p < 0.001$; **$p < 0.01$; *$p < 0.05$

Table S9: Results of regression with sensitivity in wave_t as the outcome variable using the panel on the 2020 U.S. election. Variables with t subscript are measured in each time point. Within-participant effects are difference in difference estimates of within-participant changes from time-variant variables. Between-participant effects are estimates of differences between participants from time-invariant variables.

	Election panel, DV: Sensitivity (AUC)		
	Facebook interaction	Twitter interaction	YouTube interaction
Cross-level interactions			
Mean visits on Facebook$_t$ × Facebook an important source	−0.003 (0.036)		
Mean visits on Twitter$_t$ × Twitter an important source		−0.017 (0.045)	
Mean visits on YouTube$_t$ × YouTube an important source			0.008 (0.027)
Within-participant effects			
Mean visits on Facebook$_t$	0.006 (0.011)	0.006 (0.010)	0.006 (0.010)
Mean visits on Twitter$_t$	0.000 (0.016)	0.002 (0.017)	0.000 (0.016)
Mean visits on YouTube$_t$	0.005 (0.011)	0.005 (0.011)	0.004 (0.011)
Mean visits on Instagram$_t$	0.026 (0.019)	0.025 (0.019)	0.025 (0.020)
Between-participant effects			
Mean visits on Facebook (mean$_i$)	0.005 (0.010)	0.005 (0.010)	0.005 (0.010)
Mean visits on Twitter (mean$_i$)	0.018 (0.016)	0.018 (0.016)	0.018 (0.016)
Mean visits on YouTube (mean$_i$)	−0.017 (0.014)	−0.017 (0.014)	−0.017 (0.014)
Mean visits on Instagram (mean$_i$)	0.005 (0.025)	0.005 (0.025)	0.005 (0.025)
Age	0.001 (0.000)**	0.001 (0.000)**	0.001 (0.000)**
Female	0.003 (0.012)	0.003 (0.012)	0.003 (0.012)
Education	0.013 (0.004)**	0.013 (0.004)**	0.013 (0.004)**
Ideology (conservatism high)	−0.064 (0.003)***	−0.064 (0.003)***	−0.064 (0.003)***
News interest	0.034 (0.007)***	0.034 (0.007)***	0.034 (0.007)***
Facebook an important source	−0.073 (0.025)**	−0.073 (0.025)**	−0.073 (0.025)**
Twitter an important source	0.017 (0.044)	0.017 (0.044)	0.017 (0.044)
Youtube an important source	−0.065 (0.029)*	−0.065 (0.029)*	−0.065 (0.029)*
(Intercept)	0.725 (0.037)***	0.725 (0.037)***	0.725 (0.037)***
AIC	−2789.702	−2790.278	−2789.198
BIC	−2651.760	−2652.336	−2651.256
Log Likelihood	1418.851	1419.139	1418.599
Num. obs.	2316	2316	2316
Num. groups: anon_id	487	487	487
Var: anon_id (Intercept)	0.013	0.013	0.013
Var: Residual	0.011	0.011	0.011

$^{***}p < 0.001$; $^{**}p < 0.01$; $^{*}p < 0.05$

Table S10: Results of regression with sensitivity in wave$_t$ as the outcome variable using the panel on the 2020 U.S. election. Variables with t subscript are measured in each time point. Cross-level interactions are interactions between time-variant and time-invariant variables. Within-participant effects are difference in difference estimates of within-participant changes from time-variant variables. Between-participant effects are estimates of differences between participants from time-invariant variables.

	Election panel, DV: Sensitivity (AUC)
	Main effects (no interactions)
Within-participant effects	
Self-reported Facebook use$_t$	0.006 (0.003)
Self-reported Twitter use$_t$	0.003 (0.003)
Self-reported YouTube use$_t$	0.011 (0.003)***
Between-participant effects	
Self-reported Facebook use (mean$_i$)	0.004 (0.003)
Self-reported Twitter use (mean$_i$)	−0.001 (0.003)
Self-reported YouTube use (mean$_i$)	0.002 (0.003)
Age	0.001 (0.000)***
Female	0.007 (0.008)
Education	0.013 (0.003)***
Ideology (conservatism high)	−0.062 (0.002)***
News interest	0.030 (0.005)***
Facebook an important source	−0.073 (0.017)***
Twitter an important source	0.023 (0.025)
Youtube an important source	−0.062 (0.020)**
(Intercept)	0.686 (0.028)***
AIC	−5887.802
BIC	−5751.512
Log Likelihood	2964.901
Num. obs.	4866
Num. groups: anon_id	1040
Var: anon_id (Intercept)	0.014
Var: Residual	0.011

$^{***}p < 0.001$; $^{**}p < 0.01$; $^{*}p < 0.05$

Table S11: Results of regression with sensitivity in wave$_t$ as the outcome variable using the panel on the 2020 U.S. election. Variables with t subscript are measured in each time point. Within-participant effects are difference in difference estimates of within-participant changes from time-variant variables. Between-participant effects are estimates of differences between participants from time-invariant variables.

	Election panel, DV: Sensitivity (AUC)		
	Facebook interaction	Twitter interaction	YouTube interaction
Cross-level interactions			
Self-reported on Facebook$_t$ × Facebook an important source	−0.030 (0.011)**		
Self-reported on Twitter$_t$ × Twitter an important source		0.054 (0.017)**	
Self-reported on YouTube$_t$ × YouTube an important source			0.016 (0.014)
Within-participant effects			
Self-reported Facebook use$_t$	0.009 (0.003)*	0.006 (0.003)	0.006 (0.003)
Self-reported Twitter use$_t$	0.004 (0.003)	0.001 (0.003)	0.003 (0.003)
Self-reported YouTube use$_t$	0.011 (0.003)***	0.010 (0.003)***	0.010 (0.003)***
Between-participant effects			
Self-reported Facebook use (mean$_i$)	0.004 (0.003)	0.004 (0.003)	0.004 (0.003)
Self-reported Twitter use (mean$_i$)	−0.001 (0.003)	−0.001 (0.003)	−0.001 (0.003)
Self-reported YouTube use (mean$_i$)	0.002 (0.003)	0.002 (0.003)	0.002 (0.003)
Age	0.001 (0.000)***	0.001 (0.000)***	0.001 (0.000)***
Female	0.007 (0.008)	0.007 (0.008)	0.007 (0.008)
Education	0.013 (0.003)***	0.013 (0.003)***	0.013 (0.003)***
Ideology (conservatism high)	−0.062 (0.002)***	−0.062 (0.002)***	−0.062 (0.002)***
News interest	0.030 (0.005)***	0.030 (0.005)***	0.030 (0.005)***
Facebook an important source	−0.073 (0.017)***	−0.073 (0.017)***	−0.073 (0.017)***
Twitter an important source	0.023 (0.025)	0.023 (0.025)	0.023 (0.025)
Youtube an important source	−0.062 (0.020)**	−0.062 (0.020)**	−0.062 (0.020)**
(Intercept)	0.686 (0.028)***	0.686 (0.028)***	0.686 (0.028)***
AIC	−5886.714	−5889.745	−5880.379
BIC	−5743.934	−5746.964	−5737.598
Log Likelihood	2965.357	2966.872	2962.189
Num. obs.	4866	4866	4866
Num. groups: anon_id	1040	1040	1040
Var: anon_id (Intercept)	0.014	0.014	0.014
Var: Residual	0.011	0.011	0.011

$^{***}p < 0.001$; $^{**}p < 0.01$; $^{*}p < 0.05$

Table S12: Results of regression with sensitivity in wave$_t$ as the outcome variable using the panel on the 2020 U.S. election predicted by self-reported platform use and its interaction with platform importance.

	Election panel, DV: Sensitivity (AUC)			
	Facebook interaction	Twitter interaction	YouTube interaction	Instagram interaction
Cross-level interactions				
Duration on Facebook$_t$ × Ideology	−0.000 (0.005)			
Duration on Twitter$_t$ × Ideology		−0.005 (0.007)		
Duration on YouTube$_t$ × Ideology			0.003 (0.004)	
Duration on Instagram$_t$ × Ideology				0.036 (0.013)**
Within-participant effects				
Duration on Facebook$_t$	0.004 (0.020)	0.002 (0.009)	0.002 (0.009)	0.001 (0.009)
Duration on Twitter$_t$	0.005 (0.014)	0.023 (0.028)	0.005 (0.014)	0.007 (0.014)
Duration on YouTube$_t$	−0.004 (0.008)	−0.004 (0.008)	−0.014 (0.016)	−0.005 (0.008)
Duration on Instagram$_t$	0.037 (0.022)	0.037 (0.022)	0.037 (0.022)	−0.078 (0.047)
Between-participant effects				
Duration on Facebook (mean$_i$)	0.003 (0.009)	0.003 (0.009)	0.003 (0.009)	0.003 (0.009)
Duration on Twitter (mean$_i$)	0.024 (0.017)	0.024 (0.017)	0.024 (0.017)	0.024 (0.017)
Duration on YouTube (mean$_i$)	−0.005 (0.010)	−0.005 (0.010)	−0.005 (0.010)	−0.005 (0.010)
Duration on Instagram (mean$_i$)	−0.014 (0.022)	−0.014 (0.022)	−0.014 (0.022)	−0.014 (0.022)
Age	0.001 (0.000)**	0.001 (0.000)**	0.001 (0.000)**	0.001 (0.000)**
Female	0.004 (0.012)	0.004 (0.012)	0.004 (0.012)	0.004 (0.012)
Education	0.013 (0.004)**	0.013 (0.004)**	0.013 (0.004)**	0.013 (0.004)**
Ideology (conservatism high)	−0.063 (0.003)***	−0.063 (0.003)***	−0.063 (0.003)***	−0.063 (0.003)***
News interest	0.034 (0.007)***	0.034 (0.007)***	0.034 (0.007)***	0.034 (0.007)***
Facebook an important source	−0.071 (0.025)**	−0.071 (0.025)**	−0.071 (0.025)**	−0.071 (0.025)**
Twitter an important source	0.006 (0.045)	0.006 (0.045)	0.006 (0.045)	0.006 (0.045)
Youtube an important source	−0.066 (0.028)*	−0.066 (0.028)*	−0.066 (0.028)*	−0.066 (0.028)*
(Intercept)	0.721 (0.037)***	0.720 (0.037)***	0.721 (0.037)***	0.720 (0.037)***
AIC	−2783.411	−2784.888	−2783.578	−2793.117
BIC	−2645.469	−2646.946	−2645.636	−2655.174
Log Likelihood	1415.706	1416.444	1415.789	1420.558
Num. obs.	2316	2316	2316	2316
Num. groups: anon_id	487	487	487	487
Var: anon_id (Intercept)	0.013	0.013	0.013	0.013
Var: Residual	0.011	0.011	0.011	0.011

$^{***}p < 0.001$; $^{**}p < 0.01$; $^{*}p < 0.05$

Table S13: Results of regression with sensitivity in wave$_t$ as the outcome variable using the panel on the 2020 U.S. election. Variables with t subscript are measured in each time point. Cross-level interactions are interactions between time-variant and time-invariant variables. Within-participant effects are difference in difference estimates of within-participant changes from time-variant variables. Between-participant effects are estimates of differences between participants from time-invariant variables.

	Election panel, DV: Sensitivity (AUC)			
	Facebook interaction	Twitter interaction	YouTube interaction	Instagram interaction
Cross-level interactions				
Unique hours on Facebook_t × Ideology	0.007 (0.008)			
Unique hours on Twitter_t × Ideology		−0.008 (0.016)		
Unique hours on YouTube_t × Ideology			0.003 (0.010)	
Unique hours on Instagram_t × Ideology				$0.048 (0.023)^{*}$
Within-participant effects				
Unique hours on Facebook_t	−0.010 (0.039)	0.016 (0.020)	0.015 (0.020)	0.016 (0.020)
Unique hours on Twitter_t	−0.004 (0.034)	0.026 (0.068)	−0.005 (0.034)	−0.002 (0.034)
Unique hours on YouTube_t	0.016 (0.024)	0.016 (0.024)	0.005 (0.044)	0.013 (0.024)
Unique hours on Instagram_t	0.031 (0.043)	0.030 (0.043)	0.030 (0.043)	−0.143 (0.093)
Between-participant effects				
Unique hours on Facebook (mean_i)	0.020 (0.018)	0.020 (0.018)	0.020 (0.018)	0.020 (0.018)
Unique hours on Twitter (mean_i)	0.034 (0.032)	0.034 (0.032)	0.034 (0.032)	0.034 (0.032)
Unique hours on YouTube (mean_i)	−0.028 (0.026)	−0.028 (0.026)	−0.028 (0.026)	−0.027 (0.026)
Unique hours on Instagram (mean_i)	−0.034 (0.042)	−0.034 (0.042)	−0.034 (0.042)	−0.034 (0.042)
Age	$0.001 (0.000)^{*}$	$0.001 (0.000)^{*}$	$0.001 (0.000)^{*}$	$0.001 (0.000)^{*}$
Female	0.002 (0.012)	0.002 (0.012)	0.002 (0.012)	0.002 (0.012)
Education	$0.014 (0.004)^{**}$	$0.014 (0.004)^{**}$	$0.014 (0.004)^{**}$	$0.014 (0.004)^{**}$
Ideology (conservatism high)	$-0.063 (0.003)^{***}$	$-0.063 (0.003)^{***}$	$-0.063 (0.003)^{***}$	$-0.063 (0.003)^{***}$
News interest	$0.034 (0.007)^{***}$	$0.034 (0.007)^{***}$	$0.034 (0.007)^{***}$	$0.034 (0.007)^{***}$
Facebook an important source	$-0.073 (0.025)^{**}$	$-0.073 (0.025)^{**}$	$-0.073 (0.025)^{**}$	$-0.073 (0.025)^{**}$
Twitter an important source	0.016 (0.046)	0.016 (0.046)	0.016 (0.046)	0.016 (0.046)
Youtube an important source	$-0.063 (0.028)^{*}$	$-0.063 (0.028)^{*}$	$-0.063 (0.028)^{*}$	$-0.063 (0.028)^{*}$
(Intercept)	$0.723 (0.036)^{***}$	$0.723 (0.036)^{***}$	$0.723 (0.036)^{***}$	$0.722 (0.036)^{***}$
AIC	−2798.328	−2799.254	−2798.261	−2804.171
BIC	−2660.386	−2661.311	−2660.319	−2666.228
Log Likelihood	1423.164	1423.627	1423.131	1426.085
Num. obs.	2316	2316	2316	2316
Num. groups: anon_id	487	487	487	487
Var: anon_id (Intercept)	0.013	0.013	0.013	0.013
Var: Residual	0.011	0.011	0.011	0.011

$^{***}p < 0.001$; $^{**}p < 0.01$; $^{*}p < 0.05$

Table S14: Results of regression with sensitivity in wave_t as the outcome variable using the panel on the 2020 U.S. election. Variables with t subscript are measured in each time point. Cross-level interactions are interactions between time-variant and time-invariant variables. Within-participant effects are difference in difference estimates of within-participant changes from time-variant variables. Between-participant effects are estimates of differences between participants from time-invariant variables.

	Election panel, DV: Sensitivity (AUC)			
	Facebook interaction	Twitter interaction	YouTube interaction	Instagram interaction
Cross-level interactions				
Mean visits on Facebook$_t$ × Ideology	0.004 (0.005)			
Mean visits on Twitter$_t$ × Ideology		−0.005 (0.007)		
Mean visits on YouTube$_t$ × Ideology			0.004 (0.005)	
Mean visits on Instagram$_t$ × Ideology				0.021 (0.011)
Within-participant effects				
Mean visits on Facebook$_t$	−0.010 (0.021)	0.007 (0.010)	0.006 (0.010)	0.007 (0.010)
Mean visits on Twitter$_t$	0.001 (0.016)	0.019 (0.030)	0.001 (0.016)	0.004 (0.016)
Mean visits on YouTube$_t$	0.005 (0.011)	0.005 (0.011)	−0.011 (0.021)	0.003 (0.011)
Mean visits on Instagram$_t$	0.027 (0.019)	0.025 (0.019)	0.025 (0.019)	−0.047 (0.043)
Between-participant effects				
Mean visits on Facebook (mean$_i$)	0.005 (0.010)	0.005 (0.010)	0.005 (0.010)	0.005 (0.010)
Mean visits on Twitter (mean$_i$)	0.018 (0.016)	0.018 (0.016)	0.018 (0.016)	0.018 (0.016)
Mean visits on YouTube (mean$_i$)	−0.017 (0.014)	−0.017 (0.014)	−0.017 (0.014)	−0.017 (0.014)
Mean visits on Instagram (mean$_i$)	0.005 (0.025)	0.005 (0.025)	0.005 (0.025)	0.005 (0.025)
Age	0.001 (0.000)**	0.001 (0.000)**	0.001 (0.000)**	0.001 (0.000)**
Female	0.003 (0.012)	0.003 (0.012)	0.003 (0.012)	0.003 (0.012)
Education	0.013 (0.004)**	0.013 (0.004)**	0.013 (0.004)**	0.013 (0.004)**
Ideology (conservatism high)	−0.064 (0.003)***	−0.064 (0.003)***	−0.064 (0.003)***	−0.064 (0.003)***
News interest	0.034 (0.007)***	0.034 (0.007)***	0.034 (0.007)***	0.034 (0.007)***
Facebook an important source	−0.072 (0.025)**	−0.073 (0.025)**	−0.073 (0.025)**	−0.073 (0.025)**
Twitter an important source	0.017 (0.044)	0.017 (0.044)	0.017 (0.044)	0.017 (0.044)
Youtube an important source	−0.065 (0.029)*	−0.065 (0.029)*	−0.065 (0.029)*	−0.065 (0.029)*
(Intercept)	0.725 (0.037)***	0.725 (0.037)***	0.725 (0.037)***	0.725 (0.037)***
AIC	−2786.373	−2786.985	−2786.505	−2791.009
BIC	−2648.431	−2649.042	−2648.562	−2653.067
Log Likelihood	1417.187	1417.492	1417.252	1419.505
Num. obs.	2316	2316	2316	2316
Num. groups: anon_id	487	487	487	487
Var: anon_id (Intercept)	0.013	0.013	0.013	0.013
Var: Residual	0.011	0.011	0.011	0.011

$^{***}p < 0.001$; $^{**}p < 0.01$; $^{*}p < 0.05$

Table S15: Results of regression with sensitivity in wave$_t$ as the outcome variable using the panel on the 2020 U.S. election. Variables with t subscript are measured in each time point. Cross-level interactions are interactions between time-variant and time-invariant variables. Within-participant effects are difference in difference estimates of within-participant changes from time-variant variables. Between-participant effects are estimates of differences between participants from time-invariant variables.

	Covid-19 panel, DV: Sensitivity (AUC)
	Main effects (no interactions)
Within-participant effects	
Duration on Facebook_t	−0.004 (0.006)
Duration on Twitter_t	−0.010 (0.011)
Duration on YouTube_t	0.008 (0.005)
Duration on Instagram_t	0.000 (0.017)
Between-participant effects	
Duration on Facebook (mean_i)	0.004 (0.008)
Duration on Twitter (mean_i)	0.033 (0.016)*
Duration on YouTube (mean_i)	−0.016 (0.008)
Duration on Instagram (mean_i)	0.022 (0.019)
Age	0.001 (0.000)***
Female	−0.013 (0.009)
Education	0.020 (0.003)***
Ideology (conservatism high)	−0.051 (0.002)***
News interest	0.022 (0.006)***
Social media an important source	−0.060 (0.025)*
(Intercept)	0.758 (0.029)***
AIC	−5968.866
BIC	−5821.069
Log Likelihood	3007.433
Num. obs.	4564
Num. groups: anon_id	787
Var: anon_id (Intercept)	0.013
Var: Residual	0.011

$^{***}p < 0.001$; $^{**}p < 0.01$; $^{*}p < 0.05$

Table S16: Results of regression with sensitivity in wave_t as the outcome variable using the panel on Covid-19. Variables with t subscript are measured in each time point. Within-participant effects are difference in difference estimates of within-participant changes from time-variant variables. Between-participant effects are estimates of differences between participants from time-invariant variables.

	Covid-19 panel, DV: Sensitivity (AUC)			
	Facebook interaction	Twitter interaction	YouTube interaction	Instagram interaction
Cross-level interactions				
Duration on Facebook$_t$ × Social media an important source	−0.031 (0.028)			
Duration on Twitter$_t$ × Social media an important source		0.003 (0.040)		
Duration on YouTube$_t$ × Social media an important source			−0.008 (0.024)	
Duration on Instagram$_t$ × Social media an important source				0.019 (0.065)
Within-participant effects				
Duration on Facebook$_t$	−0.003 (0.006)	−0.004 (0.006)	−0.004 (0.006)	−0.004 (0.006)
Duration on Twitter$_t$	−0.010 (0.011)	−0.010 (0.011)	−0.010 (0.011)	−0.010 (0.011)
Duration on YouTube$_t$	0.008 (0.005)	0.008 (0.005)	0.009 (0.006)	0.008 (0.005)
Duration on Instagram$_t$	0.001 (0.017)	0.000 (0.017)	−0.000 (0.017)	−0.001 (0.017)
Between-participant effects				
Duration on Facebook (mean$_i$)	0.004 (0.008)	0.004 (0.008)	0.004 (0.008)	0.004 (0.008)
Duration on Twitter (mean$_i$)	0.033 (0.016)*	0.033 (0.016)*	0.033 (0.016)*	0.033 (0.016)*
Duration on YouTube (mean$_i$)	−0.016 (0.008)	−0.016 (0.008)	−0.016 (0.008)	−0.016 (0.008)
Duration on Instagram (mean$_i$)	0.022 (0.019)	0.022 (0.019)	0.022 (0.019)	0.022 (0.019)
Age	0.001 (0.000)***	0.001 (0.000)***	0.001 (0.000)***	0.001 (0.000)***
Female	−0.013 (0.009)	−0.013 (0.009)	−0.013 (0.009)	−0.013 (0.009)
Education	0.020 (0.003)***	0.020 (0.003)***	0.020 (0.003)***	0.020 (0.003)***
Ideology (conservatism high)	−0.051 (0.002)***	−0.051 (0.002)***	−0.051 (0.002)***	−0.051 (0.002)***
News interest	0.022 (0.006)***	0.022 (0.006)***	0.022 (0.006)***	0.022 (0.006)***
Social media an important source	−0.059 (0.025)*	−0.060 (0.025)*	−0.060 (0.025)*	−0.060 (0.025)*
(Intercept)	0.758 (0.029)***	0.758 (0.029)***	0.758 (0.029)***	0.758 (0.029)***
AIC	−5962.800	−5962.267	−5961.349	−5963.325
BIC	−5808.577	−5808.044	−5807.126	−5809.102
Log Likelihood	3005.400	3005.134	3004.674	3005.662
Num. obs.	4564	4564	4564	4564
Num. groups: anon_id	787	787	787	787
Var: anon_id (Intercept)	0.013	0.013	0.013	0.013
Var: Residual	0.011	0.011	0.011	0.011

$^{***}p < 0.001$; $^{**}p < 0.01$; $^{*}p < 0.05$

Table S17: Results of regression with sensitivity in wave$_t$ as the outcome variable using the panel on the the Covid-19 pandemic. Variables with t subscript are measured in each time point. Cross-level interactions are interactions between time-variant and time-invariant variables. Within-participant effects are difference in difference estimates of within-participant changes from time-variant variables. Between-participant effects are estimates of differences between participants from time-invariant variables.

	Covid-19 panel, DV: Sensitivity (AUC)
	Main effects (no interactions)
Within-participant effects	
Unique hours on Facebook$_t$	−0.021 (0.013)
Unique hours on Twitter$_t$	−0.026 (0.024)
Unique hours on YouTube$_t$	0.026 (0.018)
Unique hours on Instagram$_t$	0.022 (0.036)
Between-participant effects	
Unique hours on Facebook (mean$_i$)	0.010 (0.016)
Unique hours on Twitter (mean$_i$)	0.065 (0.031)*
Unique hours on YouTube (mean$_i$)	−0.060 (0.023)*
Unique hours on Instagram (mean$_i$)	0.042 (0.038)
Age	0.001 (0.000)***
Female	−0.014 (0.009)
Education	0.020 (0.003)***
Ideology (conservatism high)	−0.051 (0.002)***
News interest	0.022 (0.006)***
Social media an important source	−0.061 (0.024)*
(Intercept)	0.761 (0.028)***
AIC	−5984.303
BIC	−5836.506
Log Likelihood	3015.151
Num. obs.	4564
Num. groups: anon_id	787
Var: anon_id (Intercept)	0.013
Var: Residual	0.011

$^{***}p < 0.001$; $^{**}p < 0.01$; $^{*}p < 0.05$

Table S18: Results of regression with sensitivity in wave$_t$ as the outcome variable using the panel on Covid-19. Variables with t subscript are measured in each time point. Within-participant effects are difference in difference estimates of within-participant changes from time-variant variables. Between-participant effects are estimates of differences between participants from time-invariant variables.

	Covid-19 panel, DV: Sensitivity (AUC)			
	Facebook interaction	Twitter interaction	YouTube interaction	Instagram interaction
Cross-level interactions				
Unique hours on Facebook_t × Social media an important source	−0.064 (0.053)			
Unique hours on Twitter_t × Social media an important source		0.044 (0.092)		
Unique hours on YouTube_t × Social media an important source			−0.024 (0.070)	
Unique hours on Instagram_t × Social media an important source				0.018 (0.141)
Within-participant effects				
Unique hours on Facebook_t	−0.019 (0.013)	−0.021 (0.013)	−0.021 (0.013)	−0.021 (0.013)
Unique hours on Twitter_t	−0.027 (0.024)	−0.028 (0.024)	−0.026 (0.024)	−0.026 (0.024)
Unique hours on YouTube_t	0.026 (0.018)	0.026 (0.018)	0.027 (0.018)	0.026 (0.018)
Unique hours on Instagram_t	0.024 (0.036)	0.022 (0.036)	0.021 (0.036)	0.021 (0.037)
Between-participant effects				
Unique hours on Facebook (mean_i)	0.010 (0.016)	0.010 (0.016)	0.010 (0.016)	0.010 (0.016)
Unique hours on Twitter (mean_i)	0.065 (0.031)*	0.065 (0.031)*	0.065 (0.031)*	0.065 (0.031)*
Unique hours on YouTube (mean_i)	−0.060 (0.023)*	−0.060 (0.023)*	−0.060 (0.023)**	−0.060 (0.023)*
Unique hours on Instagram (mean_i)	0.042 (0.038)	0.042 (0.038)	0.042 (0.038)	0.042 (0.038)
Age	0.001 (0.000)***	0.001 (0.000)***	0.001 (0.000)***	0.001 (0.000)***
Female	−0.014 (0.009)	−0.014 (0.009)	−0.014 (0.009)	−0.014 (0.009)
Education	0.020 (0.003)***	0.020 (0.003)***	0.020 (0.003)***	0.020 (0.003)***
Ideology (conservatism high)	−0.051 (0.002)***	−0.051 (0.002)***	−0.051 (0.002)***	−0.051 (0.002)***
News interest	0.022 (0.006)***	0.022 (0.006)***	0.022 (0.006)***	0.022 (0.006)***
Social media an important source	−0.060 (0.024)*	−0.061 (0.024)*	−0.061 (0.024)*	−0.061 (0.024)*
(Intercept)	0.761 (0.028)***	0.761 (0.028)***	0.761 (0.028)***	0.761 (0.028)***
AIC	−5979.711	−5979.599	−5978.940	−5980.236
BIC	−5825.488	−5825.376	−5824.717	−5826.013
Log Likelihood	3013.856	3013.800	3013.470	3014.118
Num. obs.	4564	4564	4564	4564
Num. groups: anon_id	787	787	787	787
Var: anon_id (Intercept)	0.013	0.013	0.013	0.013
Var: Residual	0.011	0.011	0.011	0.011

***$p < 0.001$; **$p < 0.01$; *$p < 0.05$

Table S19: Results of regression with sensitivity in wave_t as the outcome variable using the panel on the the Covid-19 pandemic. Variables with t subscript are measured in each time point. Cross-level interactions are interactions between time-variant and time-invariant variables. Within-participant effects are difference in difference estimates of within-participant changes from time-variant variables. Between-participant effects are estimates of differences between participants from time-invariant variables.

	Covid-19 panel, DV: Sensitivity (AUC)
	Main effects (no interactions)
Within-participant effects	
Mean visits on Facebook$_t$	−0.006 (0.006)
Mean visits on Twitter$_t$	−0.013 (0.011)
Mean visits on YouTube$_t$	0.014 (0.007)
Mean visits on Instagram$_t$	0.010 (0.015)
Between-participant effects	
Mean visits on Facebook (mean$_i$)	0.004 (0.009)
Mean visits on Twitter (mean$_i$)	0.030 (0.015)
Mean visits on YouTube (mean$_i$)	−0.031 (0.012)**
Mean visits on Instagram (mean$_i$)	0.048 (0.023)*
Age	0.001 (0.000)***
Female	−0.014 (0.009)
Education	0.020 (0.003)***
Ideology (conservatism high)	−0.051 (0.002)***
News interest	0.022 (0.006)***
Social media an important source	−0.060 (0.024)*
(Intercept)	0.758 (0.029)***
AIC	−5976.075
BIC	−5828.278
Log Likelihood	3011.038
Num. obs.	4564
Num. groups: anon_id	787
Var: anon_id (Intercept)	0.013
Var: Residual	0.011

$^{***}p < 0.001$; $^{**}p < 0.01$; $^{*}p < 0.05$

Table S20: Results of regression with sensitivity in wave$_t$ as the outcome variable using the panel on Covid-19. Variables with t subscript are measured in each time point. Within-participant effects are difference in difference estimates of within-participant changes from time-variant variables. Between-participant effects are estimates of differences between participants from time-invariant variables.

	Covid-19 panel, DV: Sensitivity (AUC)			
	Facebook interaction	Twitter interaction	YouTube interaction	Instagram interaction
Cross-level interactions				
Mean visits on Facebook$_t$ × Social media an important source	−0.034 (0.029)			
Mean visits on Twitter$_t$ × Social media an important source		0.016 (0.045)		
Mean visits on YouTube$_t$ × Social media an important source			−0.015 (0.032)	
Mean visits on Instagram$_t$ × Social media an important source				0.111 (0.074)
Within-participant effects				
Mean visits on Facebook$_t$	−0.005 (0.006)	−0.006 (0.006)	−0.006 (0.006)	−0.006 (0.006)
Mean visits on Twitter$_t$	−0.013 (0.011)	−0.014 (0.011)	−0.013 (0.011)	−0.012 (0.011)
Mean visits on YouTube$_t$	0.014 (0.007)	0.014 (0.007)	0.015 (0.007)*	0.014 (0.007)
Mean visits on Instagram$_t$	0.010 (0.015)	0.010 (0.015)	0.010 (0.015)	0.006 (0.015)
Between-participant effects				
Mean visits on Facebook (mean$_i$)	0.004 (0.009)	0.004 (0.009)	0.004 (0.009)	0.004 (0.009)
Mean visits on Twitter (mean$_i$)	0.030 (0.015)	0.030 (0.015)	0.030 (0.015)	0.030 (0.015)
Mean visits on YouTube (mean$_i$)	−0.031 (0.012)**	−0.031 (0.012)**	−0.031 (0.012)**	−0.031 (0.012)**
Mean visits on Instagram (mean$_i$)	0.048 (0.023)*	0.048 (0.023)*	0.048 (0.023)*	0.048 (0.023)*
Age	0.001 (0.000)***	0.001 (0.000)***	0.001 (0.000)***	0.001 (0.000)***
Female	−0.014 (0.009)	−0.014 (0.009)	−0.014 (0.009)	−0.014 (0.009)
Education	0.020 (0.003)***	0.020 (0.003)***	0.020 (0.003)***	0.020 (0.003)***
Ideology (conservatism high)	−0.051 (0.002)***	−0.051 (0.002)***	−0.051 (0.002)***	−0.051 (0.002)***
News interest	0.022 (0.006)***	0.022 (0.006)***	0.022 (0.006)***	0.022 (0.006)***
Social media an important source	−0.060 (0.024)*	−0.060 (0.024)*	−0.060 (0.024)*	−0.060 (0.024)*
(Intercept)	0.758 (0.029)***	0.758 (0.029)***	0.758 (0.029)***	0.758 (0.029)***
AIC	−5970.182	−5969.819	−5969.263	−5972.943
BIC	−5815.959	−5815.596	−5815.040	−5818.720
Log Likelihood	3009.091	3008.909	3008.632	3010.471
Num. obs.	4564	4564	4564	4564
Num. groups: anon_id	787	787	787	787
Var: anon_id (Intercept)	0.013	0.013	0.013	0.013
Var: Residual	0.011	0.011	0.011	0.011

$^{***}p < 0.001$; $^{**}p < 0.01$; $^{*}p < 0.05$

Table S21: Results of regression with sensitivity in wave$_t$ as the outcome variable using the panel on the the Covid-19 pandemic. Variables with t subscript are measured in each time point. Cross-level interactions are interactions between time-variant and time-invariant variables. Within-participant effects are difference in difference estimates of within-participant changes from time-variant variables. Between-participant effects are estimates of differences between participants from time-invariant variables.

	COVID-19 panel, DV: Sensitivity (AUC)
	Main effects (no interactions)
Within-participant effects	
Self-reported Facebook use$_t$	−0.002 (0.003)
Self-reported Twitter use$_t$	0.008 (0.003)**
Self-reported YouTube use$_t$	0.008 (0.002)***
Between-participant effects	
Self-reported Facebook use (mean$_i$)	0.001 (0.003)
Self-reported Twitter use (mean$_i$)	0.005 (0.003)
Self-reported YouTube use (mean$_i$)	−0.004 (0.004)
Age	0.001 (0.000)***
Female	−0.011 (0.008)
Education	0.019 (0.003)***
Ideology (conservatism high)	−0.051 (0.002)***
News interest	0.023 (0.005)***
Social media as an important source	−0.061 (0.022)**
(Intercept)	0.744 (0.031)***
AIC	−7314.021
BIC	−7174.813
Log Likelihood	3678.010
Num. obs.	5591
Num. groups: anon_id	971
Var: anon_id (Intercept)	0.013
Var: Residual	0.011

$^{***}p < 0.001$; $^{**}p < 0.01$; $^{*}p < 0.05$

Table S22: Results of regression with sensitivity in wave$_t$ as the outcome variable using the panel on the COVID pandemic. Variables with t subscript are measured in each time point. Within-participant effects are difference in difference estimates of within-participant changes from time-variant variables. Between-participant effects are estimates of differences between participants from time-invariant variables.

	Covid-19 panel, DV: Sensitivity (AUC)		
	Facebook interaction	Twitter interaction	YouTube interaction
Cross-level interactions			
Self-reported Facebook use$_t$ × Social media an important source	0.022 (0.013)		
Self-reported Twitter use$_t$ × Social media an important source		−0.002 (0.014)	
Self-reported YouTube use$_t$ × Social media an important source			0.011 (0.013)
Within-participant effects			
Self-reported Facebook use$_t$	−0.002 (0.003)	−0.002 (0.003)	−0.002 (0.003)
Self-reported Twitter use$_t$	0.007 (0.003)*	0.008 (0.003)**	0.007 (0.003)*
Self-reported YouTube use$_t$	0.008 (0.002)***	0.008 (0.002)***	0.008 (0.002)**
Between-participant effects			
Self-reported Facebook use (mean$_i$)	0.001 (0.003)	0.001 (0.003)	0.001 (0.003)
Self-reported Twitter use (mean$_i$)	0.005 (0.003)	0.005 (0.003)	0.005 (0.003)
Self-reported YouTube use (mean$_i$)	−0.004 (0.004)	−0.004 (0.004)	−0.004 (0.004)
Age	0.001 (0.000)***	0.001 (0.000)***	0.001 (0.000)***
Female	−0.011 (0.008)	−0.011 (0.008)	−0.011 (0.008)
Education	0.019 (0.003)***	0.019 (0.003)***	0.019 (0.003)***
Ideology (conservatism high)	−0.051 (0.002)***	−0.051 (0.002)***	−0.051 (0.002)***
News interest	0.023 (0.005)***	0.023 (0.005)***	0.023 (0.005)***
Social media an important source	−0.061 (0.022)**	−0.061 (0.022)**	−0.061 (0.022)**
(Intercept)	0.744 (0.031)***	0.744 (0.031)***	0.744 (0.031)***
AIC	−7308.101	−7305.350	−7305.916
BIC	−7162.265	−7159.514	−7160.080
Log Likelihood	3676.050	3674.675	3674.958
Num. obs.	5591	5591	5591
Num. groups: anon_id	971	971	971
Var: anon_id (Intercept)	0.013	0.013	0.013
Var: Residual	0.011	0.011	0.011

$^{***}p < 0.001$; $^{**}p < 0.01$; $^{*}p < 0.05$

Table S23: Results of regression with sensitivity in wave$_t$ as the outcome variable using the panel on the 2020 U.S. election. Variables with t subscript are measured in each time point. Cross-level interactions are interactions between time-variant and time-invariant variables. Within-participant effects are difference in difference estimates of within-participant changes from time-variant variables. Between-participant effects are estimates of differences between participants from time-invariant variables.

	Covid-19 panel, DV: Sensitivity (AUC)			
	Facebook interaction	Twitter interaction	YouTube interaction	Instagram interaction
Cross-level interactions				
Duration on Facebook$_t$ × Ideology	−0.001 (0.003)			
Duration on Twitter$_t$ × Ideology		−0.007 (0.005)		
Duration on YouTube$_t$ × Ideology			0.002 (0.003)	
Duration on Instagram$_t$ × Ideology				−0.010 (0.008)
Within-participant effects				
Duration on Facebook$_t$	0.000 (0.012)	−0.004 (0.006)	−0.004 (0.006)	−0.004 (0.006)
Duration on Twitter$_t$	−0.010 (0.011)	0.013 (0.021)	−0.010 (0.011)	−0.010 (0.011)
Duration on YouTube$_t$	0.008 (0.005)	0.008 (0.005)	0.002 (0.011)	0.008 (0.005)
Duration on Instagram$_t$	−0.000 (0.017)	0.000 (0.017)	0.000 (0.017)	0.033 (0.032)
Between-participant effects				
Duration on Facebook (mean$_i$)	0.004 (0.008)	0.004 (0.008)	0.004 (0.008)	0.004 (0.008)
Duration on Twitter (mean$_i$)	0.033 (0.016)*	0.033 (0.016)*	0.033 (0.016)*	0.033 (0.016)*
Duration on YouTube (mean$_i$)	−0.016 (0.008)	−0.016 (0.008)	−0.016 (0.008)	−0.016 (0.008)
Duration on Instagram (mean$_i$)	0.022 (0.019)	0.022 (0.019)	0.022 (0.019)	0.022 (0.019)
Age	0.001 (0.000)***	0.001 (0.000)***	0.001 (0.000)***	0.001 (0.000)***
Female	−0.013 (0.009)	−0.013 (0.009)	−0.013 (0.009)	−0.013 (0.009)
Education	0.020 (0.003)***	0.020 (0.003)***	0.020 (0.003)***	0.020 (0.003)***
Ideology (conservatism high)	−0.051 (0.002)***	−0.051 (0.002)***	−0.051 (0.002)***	−0.051 (0.002)***
News interest	0.022 (0.006)***	0.022 (0.006)***	0.022 (0.006)***	0.022 (0.006)***
Social media an important source	−0.060 (0.025)*	−0.060 (0.024)*	−0.060 (0.025)*	−0.060 (0.025)*
(Intercept)	0.758 (0.029)***	0.758 (0.029)***	0.759 (0.029)***	0.758 (0.029)***
AIC	−5957.112	−5959.906	−5957.246	−5960.494
BIC	−5802.889	−5805.683	−5803.023	−5806.271
Log Likelihood	3002.556	3003.953	3002.623	3004.247
Num. obs.	4564	4564	4564	4564
Num. groups: anon_id	787	787	787	787
Var: anon_id (Intercept)	0.013	0.013	0.013	0.013
Var: Residual	0.011	0.011	0.011	0.011

$^{***}p < 0.001$; $^{**}p < 0.01$; $^{*}p < 0.05$

Table S24: Results of regression with sensitivity in wave$_t$ as the outcome variable using the panel on the the Covid-19 pandemic. Variables with t subscript are measured in each time point. Cross-level interactions are interactions between time-variant and time-invariant variables. Within-participant effects are difference in difference estimates of within-participant changes from time-variant variables. Between-participant effects are estimates of differences between participants from time-invariant variables.

	Covid-19 panel, DV: Sensitivity (AUC)			
	Facebook interaction	Twitter interaction	YouTube interaction	Instagram interaction
Cross-level interactions				
Unique hours on Facebook$_t$ × Ideology	−0.005 (0.005)			
Unique hours on Twitter$_t$ × Ideology		−0.017 (0.011)		
Unique hours on YouTube$_t$ × Ideology			−0.002 (0.007)	
Unique hours on Instagram$_t$ × Ideology				−0.024 (0.018)
Within-participant effects				
Unique hours on Facebook$_t$	−0.001 (0.024)	−0.022 (0.013)	−0.021 (0.013)	−0.022 (0.013)
Unique hours on Twitter$_t$	−0.027 (0.024)	0.033 (0.044)	−0.026 (0.024)	−0.026 (0.024)
Unique hours on YouTube$_t$	0.027 (0.018)	0.028 (0.018)	0.034 (0.031)	0.027 (0.018)
Unique hours on Instagram$_t$	0.018 (0.037)	0.020 (0.036)	0.021 (0.036)	0.098 (0.069)
Between-participant effects				
Unique hours on Facebook (mean$_i$)	0.010 (0.016)	0.010 (0.016)	0.010 (0.016)	0.010 (0.016)
Unique hours on Twitter (mean$_i$)	0.065 (0.031)*	0.065 (0.031)*	0.065 (0.031)*	0.065 (0.031)*
Unique hours on YouTube (mean$_i$)	−0.060 (0.023)*	−0.060 (0.023)*	−0.060 (0.023)*	−0.060 (0.023)*
Unique hours on Instagram (mean$_i$)	0.042 (0.038)	0.042 (0.038)	0.042 (0.038)	0.041 (0.038)
Age	0.001 (0.000)***	0.001 (0.000)***	0.001 (0.000)***	0.001 (0.000)***
Female	−0.014 (0.009)	−0.014 (0.009)	−0.014 (0.009)	−0.014 (0.009)
Education	0.020 (0.003)***	0.020 (0.003)***	0.020 (0.003)***	0.020 (0.003)***
Ideology (conservatism high)	−0.051 (0.002)***	−0.051 (0.002)***	−0.051 (0.002)***	−0.051 (0.002)***
News interest	0.022 (0.006)***	0.022 (0.006)***	0.022 (0.006)***	0.022 (0.006)***
Social media an important source	−0.061 (0.024)*	−0.061 (0.024)*	−0.061 (0.024)*	−0.061 (0.024)*
(Intercept)	0.761 (0.028)***	0.761 (0.028)***	0.761 (0.028)***	0.761 (0.028)***
AIC	−5974.698	−5977.646	−5974.384	−5977.813
BIC	−5820.475	−5823.423	−5820.161	−5823.590
Log Likelihood	3011.349	3012.823	3011.192	3012.906
Num. obs.	4564	4564	4564	4564
Num. groups: anon_id	787	787	787	787
Var: anon_id (Intercept)	0.013	0.013	0.013	0.013
Var: Residual	0.011	0.011	0.011	0.011

$^{***}p < 0.001$; $^{**}p < 0.01$; $^{*}p < 0.05$

Table S25: Results of regression with sensitivity in wave$_t$ as the outcome variable using the panel on the the Covid-19 pandemic. Variables with t subscript are measured in each time point. Cross-level interactions are interactions between time-variant and time-invariant variables. Within-participant effects are difference in difference estimates of within-participant changes from time-variant variables. Between-participant effects are estimates of differences between participants from time-invariant variables.

	Covid-19 panel, DV: Sensitivity (AUC)			
	Facebook interaction	Twitter interaction	YouTube interaction	Instagram interaction
Cross-level interactions				
Mean visits on Facebook$_t$ × Ideology	−0.000 (0.003)			
Mean visits on Twitter$_t$ × Ideology		−0.006 (0.005)		
Mean visits on YouTube$_t$ × Ideology			0.003 (0.003)	
Mean visits on Instagram$_t$ × Ideology				−0.006 (0.008)
Within-participant effects				
Mean visits on Facebook$_t$	−0.005 (0.012)	−0.006 (0.006)	−0.006 (0.006)	−0.006 (0.006)
Mean visits on Twitter$_t$	−0.013 (0.011)	0.005 (0.019)	−0.013 (0.011)	−0.013 (0.011)
Mean visits on YouTube$_t$	0.014 (0.007)	0.015 (0.007)*	0.006 (0.013)	0.015 (0.007)*
Mean visits on Instagram$_t$	0.010 (0.015)	0.010 (0.015)	0.010 (0.015)	0.027 (0.029)
Between-participant effects				
Mean visits on Facebook (mean$_i$)	0.004 (0.009)	0.004 (0.009)	0.004 (0.009)	0.004 (0.009)
Mean visits on Twitter (mean$_i$)	0.030 (0.015)	0.030 (0.015)	0.030 (0.015)	0.030 (0.015)
Mean visits on YouTube (mean$_i$)	−0.031 (0.012)**	−0.031 (0.012)**	−0.031 (0.012)**	−0.031 (0.012)**
Mean visits on Instagram (mean$_i$)	0.048 (0.023)*	0.048 (0.023)*	0.048 (0.023)*	0.047 (0.023)*
Age	0.001 (0.000)***	0.001 (0.000)***	0.001 (0.000)***	0.001 (0.000)***
Female	−0.014 (0.009)	−0.014 (0.009)	−0.014 (0.009)	−0.014 (0.009)
Education	0.020 (0.003)***	0.020 (0.003)***	0.020 (0.003)***	0.020 (0.003)***
Ideology (conservatism high)	−0.051 (0.002)***	−0.051 (0.002)***	−0.051 (0.002)***	−0.051 (0.002)***
News interest	0.022 (0.006)***	0.022 (0.006)***	0.022 (0.006)***	0.022 (0.006)***
Social media an important source	−0.060 (0.024)*	−0.060 (0.024)*	−0.060 (0.024)*	−0.060 (0.024)*
(Intercept)	0.758 (0.029)***	0.758 (0.029)***	0.758 (0.029)***	0.758 (0.029)***
AIC	−5964.140	−5966.534	−5965.047	−5966.810
BIC	−5809.917	−5812.312	−5810.824	−5812.587
Log Likelihood	3006.070	3007.267	3006.523	3007.405
Num. obs.	4564	4564	4564	4564
Num. groups: anon_id	787	787	787	787
Var: anon_id (Intercept)	0.013	0.013	0.013	0.013
Var: Residual	0.011	0.011	0.011	0.011

$^{***}p < 0.001$; $^{**}p < 0.01$; $^{*}p < 0.05$

Table S26: Results of regression with sensitivity in wave$_t$ as the outcome variable using the panel on the the Covid-19 pandemic. Variables with t subscript are measured in each time point. Cross-level interactions are interactions between time-variant and time-invariant variables. Within-participant effects are difference in difference estimates of within-participant changes from time-variant variables. Between-participant effects are estimates of differences between participants from time-invariant variables.

References

[1] R. K. Garrett and R. M. Bond, "Conservatives' susceptibility to misinformation," *Science Advances*, vol. eabf1234, 2019.

[2] N. Macmillan and C. D. Creelman, *Detection Theory: A User's Guide*. New York: Psychology Press, 2005.

[3] G. Firebaugh, C. Warner, and M. Massoglia, *Handbook of Causal Analysis for Social Research*, ch. Fixed Effects, Random Effects, and Hybrid Models for Causal Analysis. Springer Science + Business, 2013.
